# Scale morphology and squamation pattern of *Guiyu oneiros* provide new insights into early osteichthyan body plan

**DOI:** 10.1038/s41598-019-40845-7

**Published:** 2019-03-13

**Authors:** Xindong Cui, Tuo Qiao, Min Zhu

**Affiliations:** 1 Key Laboratory of Vertebrate Evolution and Human Origins of Chinese Academy of Sciences, Institute of Vertebrate Paleontology and Paleoanthropology, Chinese Academy of Sciences, Beijing, 100044 China; 2 CAS Center for Excellence in Life and Paleoenvironment, Beijing, 100044 China; 3 University of Chinese Academy of Sciences, Beijing, 100049 China

## Abstract

Scale morphology and squamation play an important role in the study of fish phylogeny and classification. However, as the scales of the earliest osteichthyans or bony fishes are usually found in a disarticulated state, research into squamation patterns and phylogeny has been limited. Here we quantitatively describe the scale morphology of the oldest articulated osteichthyan, the 425-million-year-old *Guiyu oneiros*, based on geometric morphometrics and high-resolution computed tomography. Based on the cluster analysis of the scales in the articulated specimens, we present a squamation pattern of *Guiyu oneiros*, which divides the body scales into 4 main belts, comprising 16 areas. The new pattern reveals that the squamation of early osteichthyans is more complicated than previously known, and demonstrates that the taxa near the crown osteichthyan node in late Silurian had a greater degree of squamation zonation compared to more advanced forms. This study offers an important reference for the classification of detached scales of early osteichthyans, provides new insights into the early evolution of osteichthyan scales, and adds to our understanding of the early osteichthyan body plan.

## Introduction

Research of the scale morphology and squamation of both fossil^1–7^ and extant osteichthyans^8–14^ provides valuable information for the fish systematics and phylogeny. Previous descriptive works on early osteichthyan scale-based taxa were based on qualitative descriptions, or simple quantitative descriptions, lacking any systematic morphometric data^2,15–20^. For instance, Schultze^2^ described the scales of *Ligulalepis*, *Dialipina* and *Orvikuina*, using the ratio of height/length and free field length/total scale length^5,6,18–20^, but the peg and the anterodorsal process were subject to qualitative depictions.

Esin^18^ designed a scheme to describe the squamation pattern of the Permian actinopterygian *Amblypterina*, dividing the body into nine regions of distinct scale morphology. The scheme was successfully applied to several Middle-Late Devonian actinopterygian genera (*Donnrosenia*^21^, *Gogosardina*^22^, *Moythomasia*^20^ and *Mimipiscis*^23^). Attempts were also made to classify disarticulated scales from a number of earlier non-actinopterygian taxa using this method^5,24^. Chen *et al*.^6^ established a squamation pattern of *Andreolepis*, a late Silurian stem osteichthyan from Gotland, Sweden. They classified detached scales into 10 morphotypes, with landmark-based geometric morphometrics. Each morphotype was tentatively assigned to a specific area of the body, after the Esin’s pattern. Qu *et al*.^5^ assigned 7 morphotypes scales of *Psarolepis*, a closely related genus with *Guiyu*, to different regions of the body, based on squamation scheme of Esin^18^. Choo *et al*.^24^ described the scale morphology and squamation of *Sparalepis*, an incomplete articulated osteichthyan, from the late Silurian of Yunnan, China, still following the Esin’s pattern. However, since Esin’s squamation pattern was originally established based on a Permian actinopterygian and later affirmed by several Late Devonian actinopterygians with articulated specimens (*Moythomasia*^20^, *Gogosardina*^22^ and *Mimipiscis*^23^), it has its limitation when applying to more differentiated scales of earlier osteichthyans^5,6,24^.

The discovery of *Guiyu oneiros* firstly offered the possibility to study on the scale morphology and squamation of the late Silurian osteichthyans directly based on a complete individual. Nevertheless, the detailed research on the scales morphology and squamation of *Guiyu oneiros* has not been carried out. *Guiyu oneiros* reveals mosaic gnathostome characters, such as a primitive pectoral girdle and median fin spine as in non-osteichthyan gnathostomes, but a derived macromeric squamation as in crown osteichthyans, and substantiates the unexpected mix of postcranial features in basal sarcopterygians^25^. *Guiyu* is placed within the psarolepid-clade as a stem sarcopterygian^24,26^ or a stem osteichthyan^27,28^.

**Figure 1:**
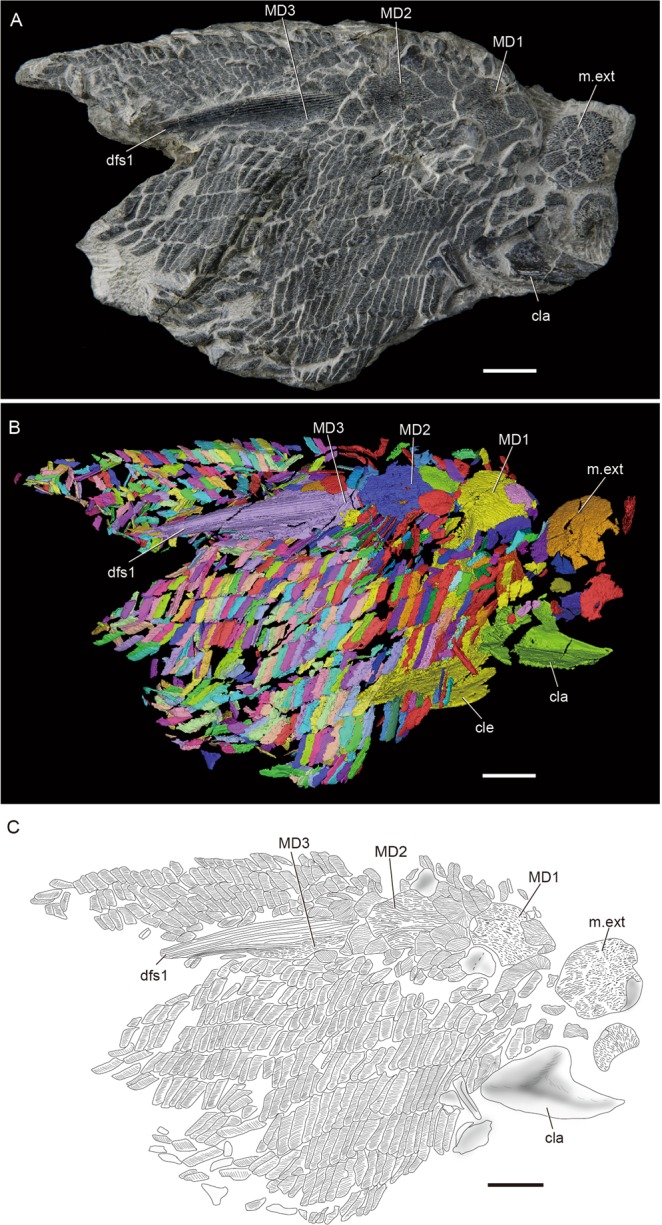
*Guiyu oneiros* Zhu *et al*., 2009. (**A**) New articulated specimen of *Guiyu oneiros* (V25047). (**B**) Three-dimensional virtual reconstruction of V25047. (**C**) Interpretative drawing of V25047. cla, clavicle; cle, cleithrum; dfs1, first dorsal fin spine; MD1–MD3, first to third median dorsal plates; m.ext, median extrascapular. Scale bar = 1 cm.

Here, by measuring the scales of *Guiyu oneiros*, the most complete articulated osteichthyan discovered from the Kuanti Formation, the late Silurian (late Ludlow) marine strata in Qujing, Yunnan Province, southwestern China^25^, we offer a new squamation pattern that would be more suitable for the classification of disarticulated rhombic scales of early osteichthyans. Besides the holotype (IVPP V15541) and IVPP V17914^29^, three new articulated partial postcraniums of *Guiyu oneiros* (IVPP V25047, V25048 and V25049.1) (Fig. 1A) were also discovered from the Kuanti Formation. Based on these articulated specimens, a new squamation pattern was established by means of quantitative methods and high-resolution computed tomography, thus rendering help to the identification of disarticulated osteichthyan scales from late Silurian to the Early Devonian.

## Results

### CT scan and three-dimensional reconstruction

Three new specimens were scanned by the 225 kV high-resolution computed tomography (CT) apparatus (developed by Institute of High Energy Physics, Chinese Academy of Sciences) at the Key Laboratory of Vertebrate Evolution and Human Origins of Chinese Academy of Sciences. More than 1000 three-dimensional models (Fig. 1B and Supplementary Videos 1, 2 and 3) of scales were reconstructed.

### Geometric morphometric analysis

**Figure 2:**
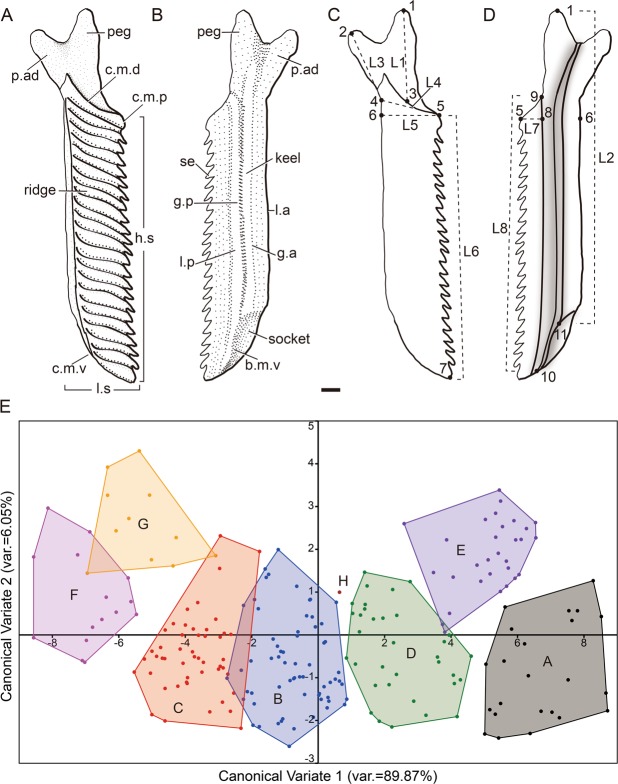
Trunk scales (M2) of *Guiyu oneiros*. (**A**,**B**) Gross anatomy of a trunk scale in crown (**A**) and basal (**B**) views. (**C**,**D**) Landmarks and length variables of a scale in crown (**C**) and basal (**D**) views. (**E**) Scatter plot of the scales in the CVA space of the first two canonical variate (discriminant) axes. b.m.v, ventral margin of base; c.m.d, dorsal margin of crown; c.m.p, posterior margin of crown; c.m.v, ventral margin of crown; g.a, anterior groove of the base; g.p, posterior groove of the base; h.s, scale height; l.a, anterior ledge; l.p, posterior ledge; p.ad, anterodorsal process; se, serration; L1–L8, first to eighth length variables; 1–11, landmarks 1–11. Landmark 1, tip of the peg; Landmark 2, tip of the anterodorsal process; Landmark 3, midpoint of the dorsal margin of crown; Landmark 4, intersection of the anterior margin of crown and the anterodorsal process; Landmark 5, intersection of the dorsal and posterior margins of crown; Landmark 6, projective point in the anterior margin of crown (or base) of Landmark 5; Landmark 7, intersection of theposterior and ventral margins of crown; Landmark 8, projective point in the posterior margin of the base of Landmark 5; Landmark 9, intersection of the posterior and dorsal margins of base; Landmark 10, intersection of the posterior and ventral margins of base; Landmark 11, the intersection of the keel and the ventral margin of base (or the rim of the socket). L1, the length of the peg; L2, the length of the keel; L3, the length of the anterodorsal process; L4, the length of scale in the longitudinal body axis; L5, the length of the scale; L6, the length of the posterior margin of crown (or the height of the scale); L7, the length of the concealed field; and L8, the length of the posterior margin of base. Scale bar = 0.5 mm.

11 landmarks (Fig. 2C) were selected on the scales of *Guiyu oneiros*, and 8 length variables (Fig. 2D) were selected for the morphometric analysis. Length 1: the length of the peg; Length 2: the length of the keel; Length 3: the length of the anterodorsal process; Length 4: the length of scale in the longitudinal body axis; Length 5: the length of the scale; Length 6: the length of the posterior margin of crown (or the height of the scale); Length 7: the length of the concealed field; and Length 8: the length of the posterior margin of base. 226 intact CT models of scales were measured to get their length variables (Supplementary Table 1).

Based on these data, a cluster analysis was performed to define scale assemblages. We first analyzed dependence of the 8 length variables. Since Lengths 2, 6 and 8 have a strong correlation, Length 6 was selected as it is the easiest one to measure. Similarly, Lengths 5 and 7 have a strong correlation, and the former one was selected.

Then, a Q-type cluster analysis was carried out based on 5 length variables (Lengths 1, 3, 4, 5 and 6) to analyze 226 scales. We chose the squared Euclidean distance, which is frequently used as the measure method, and between-groups linkage in the cluster analysis. As the 5 length variables have the same dimension, there was no need to standardize them. We tried to classify the scales into 7, 8 and 9 groups, and compared them with the fossils. The scheme of 8 groups was chosen for the higher correspondence between the scatter plots (Supplementary Fig. 1) and the distribution of the scales.

The scatter plots indicate that Lengths 1, 3 and 6 differentiate Morphotypes 1, 2, 3 and 8 (Supplementary Fig. 1A,B), and Lengths 4, 5 and 6 distinguish Morphotypes 6, 10, 11 and 12 (Supplementary Fig. 1C,D).

To test the cluster analysis result, we conducted a canonical variate analysis (CVA). The classification result of CVA indicated that the accuracy rate of previous cluster analysis result is 94%. Then, some scales were adjusted to another group because of the classification result of CVA. We carried out a CVA of the new data. The accuracy rate of the classification result enhanced to 96.04%. As such, the final cluster analysis result was shown by a scatter plot of the 8 groups scales (A, 23 scales; B, 64 scales; C, 49 scales; D, 37 scales; E, 27 scales; F, 15 scales; G, 10 scales; H, 1 scale) in the CVA space of the first two canonical variate (discriminant) axes (Fig. 2E).

### Scale morphology of *Guiyu oneiros*

**Figure 3:**
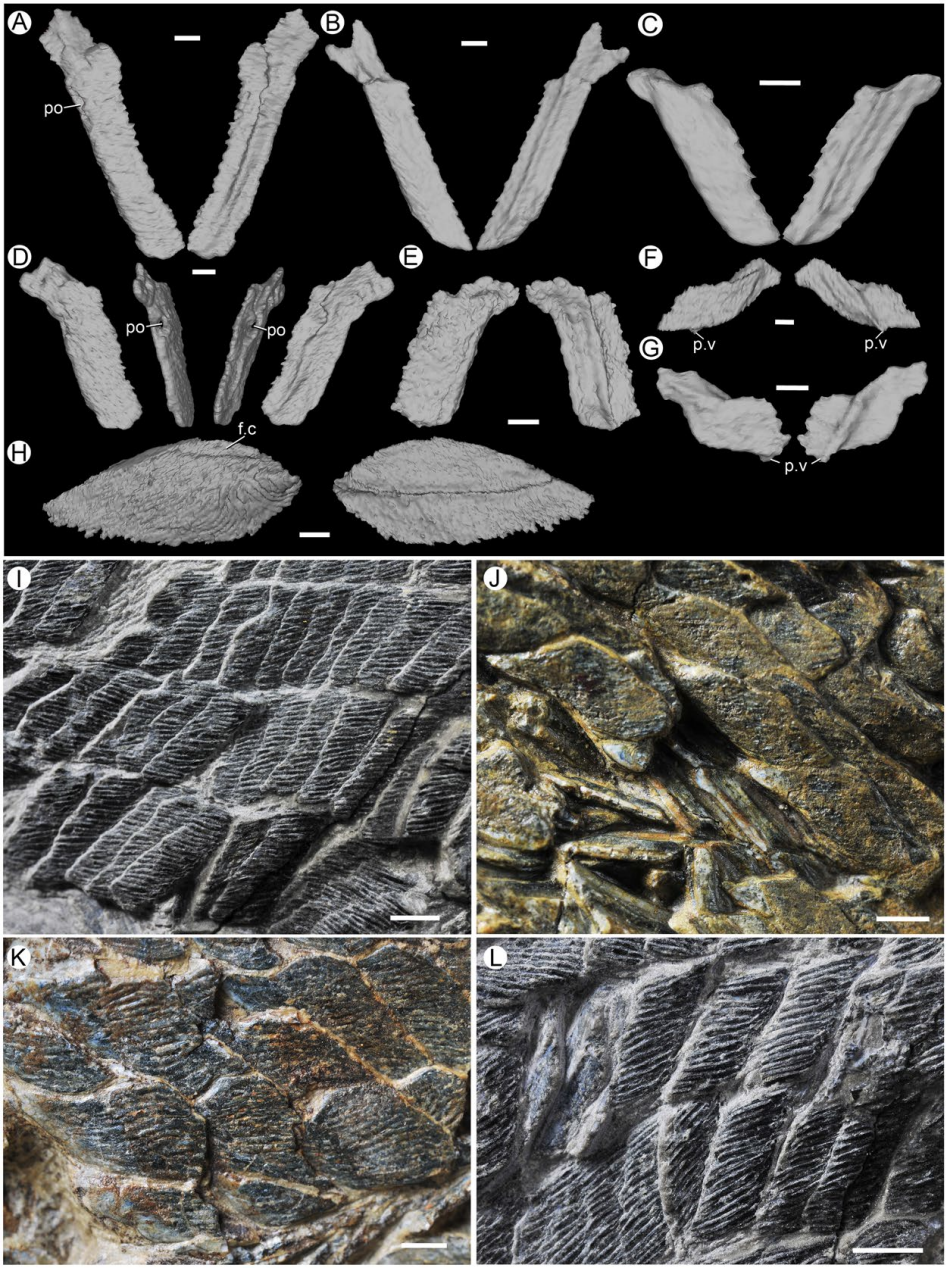
Scales of *Guiyu oneiros*. (**A**–**H**) Three-dimensional virtual reconstruction of scales in crown and basal views. (**A**–**D**) V25047. Morphotype 1 (**A**), Morphotype 2 (**B**), Morphotype 6 (**C**), Morphotype 3 (**D**). (**E**) Morphotype 3, V25048. (**F**) Morphotype 10, V25049.1. (**G**) Morphotype 11, V25047. (**H**) Morphotype 13, V25049.1. (**I**–**L**) Close-up of part of the trunk showing different scale morphotypes. (I) Morphotype 3, V25047. (**J**) Morphotype 4, V15541. (**K**) Morphotype 8, V15541. (**L**) Morphotype 6, V25047. f.c, concealed field; p.v, ventral process; po, pore of lateral line). Scale bar = 1 mm (A–H); Scale bar = 2 mm (**I**–**L**).

The scales are thin and lack a neck separating the crown and the base, unlike that in *Psarolepis*^5^. The rhomboid crown (Fig. 2A) bears no pores on the surface, and is ornamented with linear ganoid ridges that terminate with up to 30 posterior serrations. The crown is elongated, extending beyond the base posteriorly. The thin base (Fig. 3A–G) bears a weakly developed keel between prominent anterior and posterior ledges, forming two shallow grooves.

**Figure 4:**
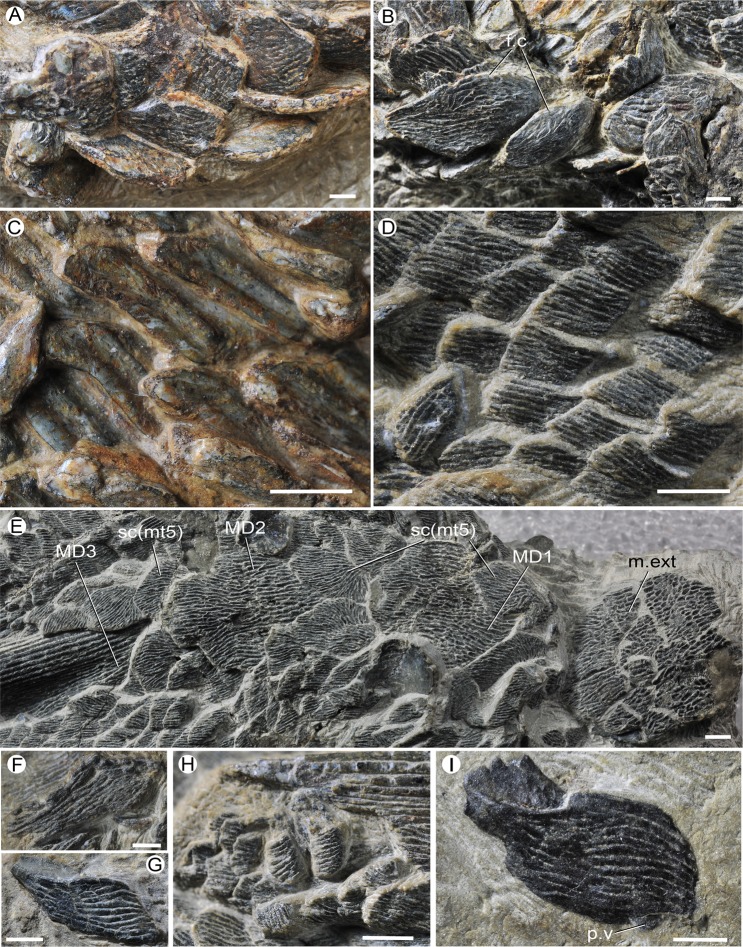
Scales of *Guiyu oneiros*. (**A**–**C**) V15541. Morphotype 12 (A), Morphotype 13 (**B**), Morphotype 7 (**C**). (**D**) Morphotype 9, V17914. (**E**) Morphotype 5 and three median dorsal plates in crown view of V25047. (**F**) Lepidotrichia, V17914. (**G**) Morphotype 10, V25049.2. (H) Basal scales of pelvic fin, V17914. (**I**) Morphotype 11, V25049.3. f.c, consealed field; MD1–MD3, first to third median dorsal plates; m.ext, median extrascapular; p.v, ventral process; sc (mt5), scale of Morphotype 5. Scale bar = 2 mm.

**Figure 5:**
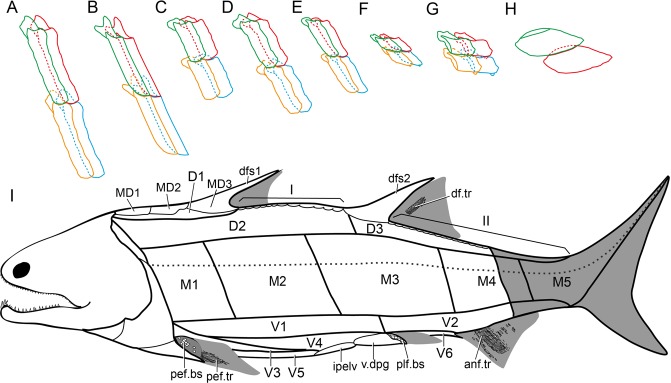
Squamation of *Guiyu oneiros*. (**A**–**H**) Articulation models of various scale morphotypes. (**A**) Morphotype 1. (**B**) Morphotype 2. (**C**) Morphotype 3. (**D**) Morphotype 8. (**E**) Morphotype 6. (**F**) Morphotype 10. (**G**) Morphotype 11. (**H**) Morphotype 13. (**I**) Squamation model of *Guiyu oneiros*. anf.tr, lepidotrichia of anal fin; D1–D3, Area D1–Area D3; df.tr, lepidotrichia of dorsal fin; dfs1, first dorsal fin spine; dfs2, second dorsal fin spine; ipelv, interpelvic plate; M1–M5, Area M1–Area M5; MD1–MD3, first to third median dorsal plates; pef.bs, basal scales of pectoral fin; pef.tr, lepidotrichia of pectoral fin; pelf.bs, basal scales of pelvic fin; V1–V6, Area V1–Area V6; v.dpg, ventral lamina of dermal pelvic girdle; I, II, areas of dorsal median ridge scales.

Although the scale articulations (pegs, sockets, anterodorsal and ventral processes) show a large variation in different morphotypes (Figs 3, 4), the flank scales share the same articulation model (Fig. 5A–G). Generally, the peg of scale wedges into the socket of the scale above, and the anterodorsal process and the concealed field of scale are covered by the scale in front (Fig. 5A–E). Some flank scales have a ventral process, that wedges under the dorsal margin of the scale below (Fig. 5F,G). The scales adjacent to the median line lack these articulations, only having a concealed narrow field, covered by the frontal scale, along the anterodorsal margin of the crown.

Lateral flank scales (Fig. 1) are rhomboid with different height/length ratios, whereas unpaired median scales (Fig. 1) are symmetrical or nearly symmetrical. According to the cluster analysis, 226 flank scales, are grouped into 8 morphotypes (Fig. 2E). In addition, 5 other morphotypes are recognized based on the squamation of the holotype^25^ and V17914^29^.

Morphotype 1 (Fig. 3A, Table 1 and Supplementary Fig. 2): Scales of roughly rectangular shape in crown view, become narrower slightly from dorsal to ventral. Each scale bears 25–35 ridges on the crown, and the serrations are indistinct. The concealed field occupies average 38% of the scale length. The concave dorsal margin of the crown, corresponding with the convex ventral margin of the crown, forming the articulation, similar to that of *Sparalepis*^24^. The anterior and posterior margins are slightly bent, like two parallel sigmoid curves.

**Table 1:** Measurement of the scales of *Guiyu oneiros*.

Morpho-type	Area	Ridge	L5 Mean (mm)	L5 SD	L6 Mean (mm)	L6 SD	L6/L5	(L6/L5) SD	L1/L6	(L1/L6) SD	L3/L6	(L3/L6) SD
1	M1	25–35	1.75	0.18	8.25	0.51	4.75	0.55	0.25	0.06	0.16	0.04
2	M2	25~30	2.03	0.18	7.17	0.41	3.55	0.34	0.32	0.05	0.29	0.04
3	M3	10~25	1.76	0.27	5.24	0.46	3.06	0.63	0.12	0.07	0.15	0.07
6	D2	15~20	1.48	0.26	3.90	0.38	2.73	0.65	0.08	0.09	0.14	0.09
8	V1	15~25	1.95	0.28	6.55	0.40	3.41	0.53	0.18	0.07	0.18	0.05
10	V3	5~10	1.42	0.27	2.27	0.52	1.66	0.44	0.10	0.13	0.30	0.21
11	V4	~10	2.37	0.39	2.55	0.50	1.11	0.32	0.31	0.07	0.36	0.20
12	V5	10~15	3.35	0.00	5.66	0.00	1.69	0.00	0.14	0.00	0.14	0.00

L1, the length of the peg; L3, the length of the anterodorsal process; L5, the length of the scale; L6, the length of the posterior margin of crown (or the height of the scale); SD, standard deviation.

In basal view, the base bears a developed posterior ledge, that becomes less distinct from dorsal to ventral. The keel is almost indiscernible.

The peg is broad-based, matching the broad socket of the neighbouring scale. The blunt anterodorsal process extends out anterodorsally, and forms an irregular trapezoid together with the peg.

Morphotype 2 (Fig. 3B, Table 1 and Supplementary Figs 2 and 3A,F): In crown view, the scales are roughly rhombic in shape. They are lower than the scales of Morphotype 1. There are 25–30 parallel ganoid ridges on the crown, terminating in the same number of serrations along the posterior margin. The ridges are smooth and devoid of accessory ridges. The concealed field is average 34% of the scale length. The dorsal margin of the crown is obviously concave, corresponding with the convex ventral margin of the crown, effectively forming the articulation.

In basal view, the keel is weakly developed, flanked by a shallow groove on each side. The anterior and posterior margins of the base are straight and roughly parallel, with robust ledges.

The anterodorsal process is extremely prominent with a sharp apex. The pennant-shaped peg is high and the socket is long and deep. The anterodorsal process and peg form a chevron-shaped concave.

Morphotype 3 (Fig. 3D,E,I, Table 1 and Supplementary Figs 2 and 3C,E): Scales have an average height/length ratio of 3.06, lower than those of Morphotype 2. 10–25 ganoid ridges are present on the crown. The concealed field constitutes 35% of the scale length. The crown has a concave dorsal margin and a convex ventral margin, as in Morphotypes 1 and 2. In basal view, the posterior ledge and grooves are developed. The anterodorsal process is more prominent than the peg. Like Morphotype 1, anterodorsal process is confluent with the broad peg.

Morphotype 4 (Fig. 3J and Supplementary Fig. 3G): The small scales are rhombic in shape, having a height/length ratio of about 2.0. Bearing 10–15 parallel ganoid ridges, the crown processes a straight posterior margin, a gently concave dorsal margin and a convex ventral margin. In basal view, the keel, anterior and posterior ledges are well-developed. The anterodorsal process is very prominent. The broad-based peg and the deep socket are triangular.

Morphotype 5 (Fig. 4E): Around the median dorsal plates, there are three pairs of large oval scales (about 8 mm in length, about 5 mm in width) and several pairs of small oval scales (5 mm in length, 3 mm in width) with a posterior apex. The crown is ornamented by ganoid ridges like other morphotypes.

Morphotype 6 (Fig. 3C,L, Table 1 and Supplementary Fig. 2): Scales of roughly rhomboid shape have an average height/length ratio of 2.34. The crown processes 15–20 ridges. The concealed field extends 35% of the scale length. In basal view, the anterior and posterior ledge is developed. The scales have a less developed keel between two shallow grooves. A pronounced anterodorsal process looks like a slender beak. However, the broad peg, merging with the anterodorsal process, is very low. To accommodate the confluent anterodorsal process and peg, the socket is broad and shallow.

Morphotype 7 (Fig. 4C): These scales are similar to Morphotype 3 in shape, and have a height/length ratio of 1.80. The crown bears 5–15 ganoid ridges, less than those of Morphotype 3. The posterior ledge is as developed as the anterior ledge, but they are less developed than the keel, forming two distinct grooves. The anterodorsal process is rounded and confluent with a low and base-broad peg.

Morphotype 8 (Fig. 3K, Table 1 and Supplementary Fig. 2): The scales are generally similar to Morphotype 3 in crown view, but they are higher and have a larger height/length ratio. The crown bears 15–25 ridges. The concealed field makes up average 38% scale length. The base bears a pronounced posterior ledge and two shallow grooves. The ligulate anterodorsal process stretches out, merging with the less developed peg. The socket is broad and shallow.

Morphotype 9 (Fig. 4D and Supplementary Fig. 3D): The small scales are rhombic in crown shape. They have a height/length ratio varying from about 1.0 to 2.0. There are 5–10 ganoid ridges on the crown. The articulations, especially the peg, are weak.

Morphotype 10 (Figs 3F, 4G, Table 1 and Supplementary Fig. 2): They are the lowest scales among all the morphotypes, with an average height/length ratio of 1.66. About 5–10 ganoid ridges are present on the elongated crown, much fewer than other morphotypes. The concealed field accounts for 37% of the scale length. A pronounced posterior ledge of the base stretches out the crown ventrally, and wedges under the dorsal margin of the neighbouring scale, as in *Psarolepis*^5^. The base bears a short but protruding keel, flanked by deep grooves. These scales have considerably prominent articulations, with the combined peg and anterodorsal process extending beyond the crown.

Morphotype 11 (Figs 3G, 4I, Table 1 and Supplementary Fig. 2): In crown view, the scales are rhombic with a height of 1.04 times the length. About 10 ganoid ridges are visible on the crown. The concealed field makes up 35% of the scale length. There is no definite boundary between the anterior and ventral margins of the crown, forming a long convex margin. The conspicuous posterior ledge of the base stretches out the crown ventrally as in Morphotype 10. The keel is so faint that the base is flat without any grooves. These scales bear well-developed confluent articulations. And the enlarged anterodorsal process is more prominent than the blunt peg.

Morphotype 12 (Fig. 4A, Table 1 and Supplementary Figs 2 and 3B): The large scales are symmetrical or near symmetrical, with a roughly oval shape. There are more than six pairs of oval scutes between the pectoral and pelvic regions^29^. Unlike most flank scales, the base of these scales is almost smooth, with no keel or ledge.

Morphotype 13 (Figs 3H and 4B): As reconstructed previously, posterior to the interpelvic plate lie a series of small scales that probably framed the cloacal opening^29^. Immediately posterior to these small scales are six pairs of scutes arranged with the long axis projecting posterolaterally. The anterior five pairs are narrow with the long axis being about 3.5 times longer than the short axis. The final pair is broader, with the long axis about twice the length of the short axis. Each of these scales may have a conspicuous keel but lack anterior or posterior ledge of the base. A narrow concealed field lies along the anterodorsal margin of the crown.

Basal scales of fins (Fig. 4H): The small rhombic basal scales of pectoral and pelvic fins are dramatically smaller than the other morphotypes. In the crown view, there are about 10 parallel ganoid ridges, that are perpendicular to the anterior margin. The base is unknown as it is not exposed. The peg is not preserved. These scales cover the pectoral and pelvic peduncle, and could be compared to the pectoral and caudal peduncle scales of *Andreolepis* that are small and elongated^6^. It corroborates the assignment of “subtype 7” of *Psarolepis* to the fin peduncles by Qu *et al*.^5^.

Lepidotrichia or fin rays (Fig. 4F): The lepidotrichia are elongated rhomboid and of the same shape and size. They corroborate that the elongated scales of *Andreplepis hedei*^30^ and “subtype 3” in *Psarolepis*^5^, may belong to the lepidotrichia of fin lobes^31,32^.

Lateral line scales (Fig. 3B,D): Some flank scales, with a lateral line canal, are discernable in the articulated material, revealing that the lateral line runs along scales of the middle flank row. The lateral line canal enters the scale anteriorly at the anterior junction of the base with the crown and exits between the posterior ledge of the base and the crown. The foramen is about 1 mm in diameter. These scales, being assigned to Morphotypes 1–4, have a wide range of height/length ratio. The anterior margin of the crown and the posterior ledge both have an incurve at the level of the sensory canal. The lateral line canal is invisible on the crown surface, unlike some crown gnathostomes^33^ whose lateral line canal has openings on the crown^34^.

### Median dorsal plates of *Guiyu oneiros*

The new articulated specimen (V25047) presents three median dorsal plates (MD, Figs 1 and 4E) behind the median extrascapular, reaffirming the revised restoration of *Guiyu oneiros*^29^. In external view (Figs 1 and 4E), each MD plate bears more than 20 longitudinal wavy ridges composed of numerous small elongated tubercles. The internal surface is smooth except a keel as described in the holotype.

The first and second MD plates (Figs 1 and 4E) are rhombic. The first (14 mm in length, 11 mm in width) is shorter than the latter (17 mm in length, 11 mm in width). The anterior and posterior margins of the first MD plate are convex with a blunt bulge, however, the second MD plate has a convex anterior margin and a concave posterior margin, as in the previous description. Both the plates are covered by a pair of larger oval scales and a pair of smaller oval ridge scales with a posterior point. By comparison, *Sparalepis* lacks these paired ridge scales to overlap the MD plates^24^.

The third MD plate (Figs 1 and 4E) bears a spine, which is the first dorsal fin spine. The hollow spine, 39.2 mm long, is ornamented by parallel linear ridges and becomes thinner posteriorly. The oval base of the third MD plate is 26.2 mm long and 10.5 mm wide, and larger than the first two MD plates.

### Squamation model of *Guiyu oneiros*

All the scales come from articulated or nearly articulated specimens of the same species. The scale height/length ratio of the holotype is considerably smaller than that of the other four specimens, which might reflect the allometric growth. Alternatively, the greater height/length ratio of the scales on speimens IVPP V17914, V25047, V25048 and V25049.1 may indicate the presence of a second species of *Guiyu*.

Based on the distribution of various morphotypes in *Guiyu oneiros*, we propose a new squamation pattern (Fig. 5I) of osteichthyans, which is more inclusive than the pattern provided by Esin^18^. According to the new pattern, the body scales of *Guiyu oneiros* could be roughly divided into 4 main belts: the dorsal, middle, ventral and unpaired-scale belts. The dorsal belt is further divided into 3 areas, comprising up to 6 horizontal rows of scales. The middle belt contains 5 areas, comprising up to 4 horizontal rows of scales. The ventral belt contains about 5 horizontal rows scales, that are assigned to 6 areas. The unpaired-scale belt, composed of 2 areas (symbolized by I and II), is located along the median dorsal line of the body.

Dorsal belt (Fig. 5I). The area D1 (Morphotype 5) is situated around the three MD plates; the area D2 (Morphotype 6) extends between the post-temporal and the base of the second dorsal fin spine longitudinally (about 30 rows of scales); and the area D3 (Morphotype 7) is situated immediately posterior to the area D2, extending to the posterior edge of the caudal peduncle.

Middle belt (Fig. 5I). The area M1 (Morphotype 1) extends from the median extrascapular to the base of the first dorsal fin spine longitudinally (about 10 rows of scales); the area M2 (Morphotype 2) is located immediately posterior to the area M1, terminating at the anterior edge of the pelvic girdle longitudinally (about 14 rows of scales); the area M3 (Morphotype 3) is located immediately posterior to the area M2, ending at the anterior edge of the pelvic girdle longitudinally (about 14 rows of scales); the area M4 (Morphotype 4) is located immediately posterior to the area M3, ending at the posterior edge of the pelvic girdle longitudinally; and the area M5 is located immediately posterior to the area M4, ending at the posterior edge of the pelvic girdle longitudinally, but the this part is not preserved on the fossil.

Ventral belt (Fig. 5I). The area V1 (Morphotype 8) is located under the areas M1, 2 and 3, ending at the posterior edge of the pelvic girdle; the area V2 (Morphotype 9) is situated immediately posterior to the area V1, and extends to the posterior edge of the caudal peduncle longitudinally; the area V3 (Morphotype 10) is located immediately posterior to the pectoral girdle; the area V4 (Morphotype 11) is situated immediately lower to the area V1 and posterior to the area V3; the area V5 (Morphotype 12) is near the ventral median line between the pectoral girdle and the interpelvic plate; and the area V6 (Morphotype 13) is close to the ventral median line as the area V5, but it is located between the interpelvic plate and the anal fin.

Unpaired-scale belt (Fig. 5I). The area I is located at the median dorsal line between the first and the second median dorsal fins; and the area II is located at the median dorsal line between the second median dorsal fin and the tail fin. It is roughly equal to the area II of the pattern designed by Esin^18^. However, as the caudal fin is not preserved, we do not extend this area to the caudal fin as in the pattern designed by Esin^18^.

### Phylogenetic results

To explore the interrelationship of early osteichthyans, we conducted phylogenetic analyses using a dataset with 347 characters and 104 taxa. The dataset was based on that of Choo *et al*.^24^ with the addition of 6 characters (characters 337–342) from Lu *et al*.^27^ and 5 new characters (character 343, keel of scale; character 344, posterior ledge or secondary keel of scale; character 345, anteroventral process of scale; character 346, peg and anterodorsal process of scale; character 347, ventral process of scale). All characters were treated as unordered and weighted equally.

Two agnathan taxa (Galeaspida and Osteostraci) were set as the outgroup. Our analysis generated 1248 trees of 979 steps (CI = 0.3790; HI = 0.6210; RI = 0.8052; RCI = 0.3051), that are summarized as a strict consensus tree (SCT) (Supplementary Fig. 4A) and a 50% majority-rule consensus tree (MCT) (Supplementary Fig. 4B).

*Guiyu*, with other members of the psarolepid cluster (*Psarolepis*, *Achoania*, *Sparalepis*)^24,35,36^ is recovered as a stem sarcopterygian (Supplementary Fig. 4), consistent with the phylogeny of Choo *et al*.^24^ and Qiao *et al*.^26^, but different from the result placing *Guiyu* within stem osteichthyans^27,28^. Both the SCT and MCT place *Lophosteus*, *Dialipina* and *Ligulalepis* as the stem osteichthyans (Supplementary Fig. 4).

## Discussion

### Early evolution of rhombic scales

**Figure 6:**
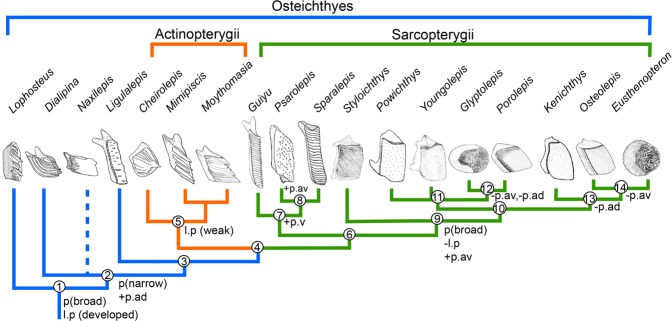
Summary phylogeny of early osteichthyans showing the scale transformation in posterior ledge, peg-socket articulation and anterodorsal process. This cladogram is simplified from the strict consensus tree of the 1248 most parsimonious trees (Supplementary Fig. 4). The scale drawing sources: *Lophosteus*^17^, *Dialipina*^2^, *Naxilepis*^41^, *Ligulalepis*^19^, *Cheirolepis*^40^, *Mimipiscis*^23^, *Psarolepis*^5^, *Sparalepis*^24^, and the rest^42^. l.p (developed), developed posterior ledge; l.p (weak), weak posterior ledge; −l.p, posterior ledge absent; p (broad), broad peg; p (narrow), narrow peg; +p.ad, anterodorsal process; −p.ad, anterodorsal process absent; +p.av, anteroventral process; −p.av, anteroventral process absent; +p.v, ventral process.

Conventionally, the rhombic scales comprise ganoid and cosmoid scales^7,37^. Schultze^37^ proposed that ganoid and cosmoid scales evolved from primitive *Lophosteus-*like scales. As our phylogenetic analysis shows (Supplementary Fig. 4), *Lophosteus*, *Dialipina*, *Naxilepis* and *Ligulalepis* are placed as stem osteichthyans, while *Guiyu*, *Sparalepis* and *Psarolepis* are resolved as stem sarcopterygians (Fig. 6, node 7).

The posterior ledge of the scale is well developed in stem osteichthyans^2,4,38^ (Fig. 6, nodes 1, 2 and 3), but vestigial in actinopterygians^20,23,38–40^ (Fig. 6, node 5), indicating a possible synapomorphy of that clade. In stem sarcopterygians (Fig. 6, node 7), the posterior ledge of scale is also developed as in stem osteichthyans. However, the posterior ledge is absent in crown sarcopterygians (Fig. 6, node 9), which suggests that this may be a synapomorphy of this cluster.

The peg-socket articulation is present in rhombic scales of stem osteichthyans as for crown osteichthyans, such as *Amblypterina*^18^ and *Gogosardina*^22^ (Fig. 6). The peg first appears at node 1, and becomes slender and developed at node 2 except *Naxilepis*^41^. The scale of *Naxilepis* has a low and broad-based peg, but this may only represent the scale from the posterior part of body considering its small height/length ratio. The peg is narrow in actinopterygians (Fig. 6, node 5), in contrast to broad in crown sarcopterygians (Fig. 6, node 9). Flank scales of *Guiyu* bear a slender peg (Figs 2A,B and 3B), whose size and shape are variable from different body regions. The flank scales from the anterior part of *Guiyu* display a developed peg (Figs 2 and 3B) with a sharp apex, as in stem osteichthyans^2,4,38^ and actinopterygians^20,23,38,39^, while the flank scales from the posterior part show a lower and blunt peg (Figs 3E and 4D).

The anterodorsal process occurs at node 2, and is developed in stem osteichthyans^2,4,38^, actinopterygians^20,23,38,39^ (Fig. 6, node 5) and stem sarcopterygians (Fig. 6, node 7). The anterodorsal process becomes weaker gradually in primitive crown sarcopterygians and disappears in derived sarcopterygians (Fig. 6, nodes 12, 14). The condition that the anterodorsal process is separated from the peg, creating a chevron shaped configuration, is shared by stem osteichthyans^2,4,38^, actinopterygians^20,23,38,39^ and *Guiyu*, whose anterodorsal process and peg are both developed.

Some early osteichthyans have ventral and anteroventral processes for the articulation of scales. The ventral process, which is the extension of the posterior ledge, has not been described till this study. Almost all flank scales of *Psarolepis* have this articulation, while only the scales from the ventral belt of *Guiyu* bear the ventral process (Fig. 3F,G and 4I), but less developed. Accordingly, the ventral process may be a synapomorphy of psarolepids (Fig. 6, node 7). The anteroventral process is present in some *Psarolepis* scales as in few crown sarcopterygians^42,43^ (Fig. 6, node 9), however, it is absent in *Guiyu* scales. Hence that may be the result of parallel evolution. The more derived sarcopterygians also lack the anteroventral process (Fig. 6, nodes 12 and 14).

### Squamation pattern of gnathostomes

**Figure 7:**
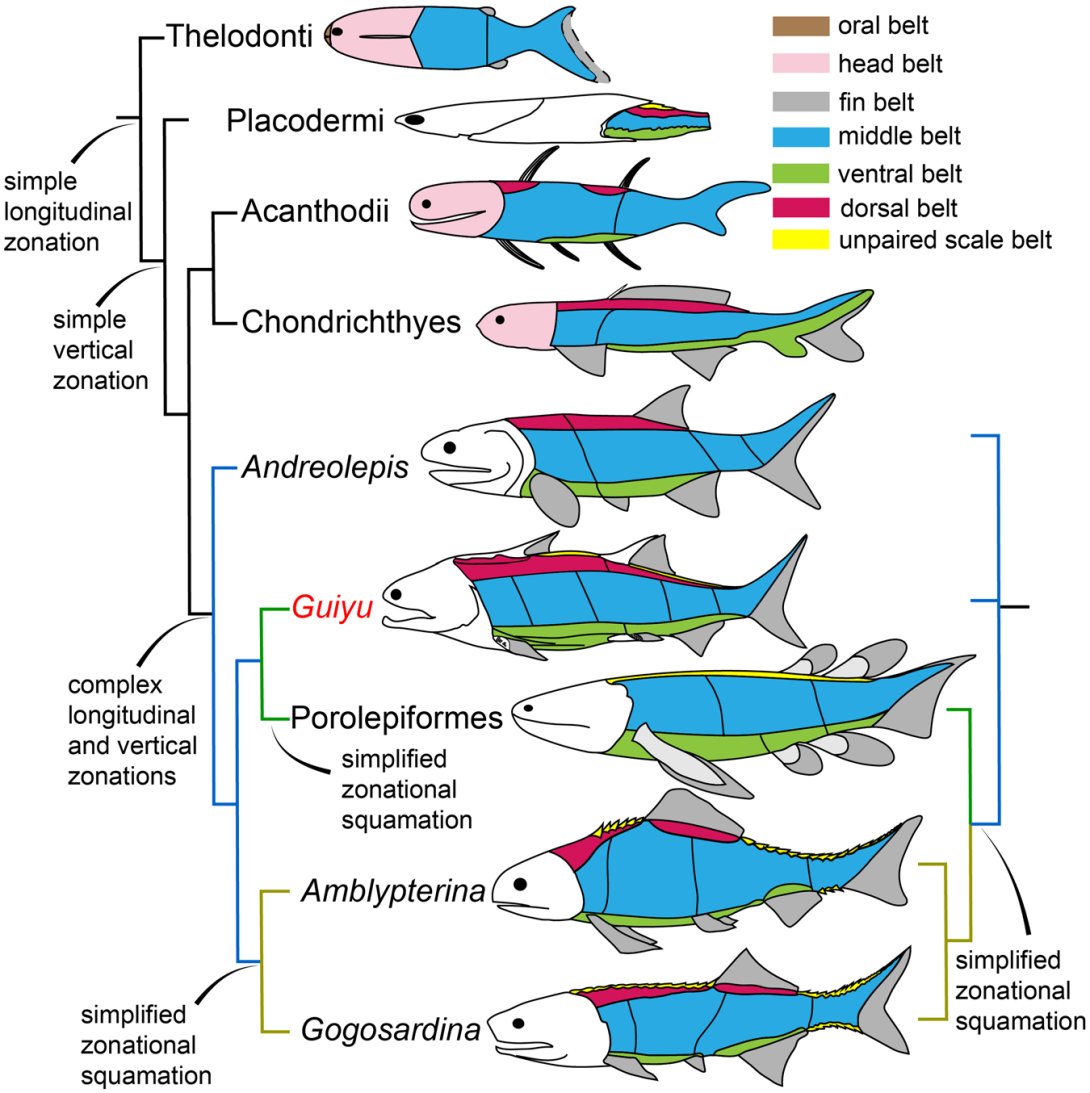
Summary phylogeny showing the squamation evolution of early osteichthyans. Taxon sources: Thelodonti^44^, Placodermi^48^, Acanthodii^49^, Chondrichthyes^50^, *Andreolepis*^6^, Porolepiformes^51^, *Amblypterina*^18^ and *Gogosardina*^23^. b.d, dorsal belt; b.h, head belt; b.m, middle belt; b.o, oral belt; b.up, unpaired scale belt; b.v, ventral belt.

The squamation pattern in agnathans^44–47^ reveals a longitudinal zonation, however in a low differentiation. The vertical zonation is widely shared by gnathostomes, so it may be a synapomorphy of gnathostomes. However, the squamation of the non-osteichthyan gnathostomes has a low zonal differentiation. The squamation of placoderms (Fig. 7), e.g. *Sigaspis lepidopbora*^48^, is composed of dorsolateral, lateral and ventrolateral scales, which is similar to that of *Guiyu*, but less complex. *Poracanthodes menneri*, as an acanthodian, has a squamation (Fig. 7) that comprises 5 areas (head, body, around the dorsal fin-spine, behind and around the anal fin-spine)^49^. The squamation of Xenacanthida (conventionally defined chondrichthyans)^50^ comprises the head, dorsal, middle and ventral belts.

In the taxa close to crown osteichthyan node, the squamation shows a further longitudinal differentiation, as shown by the complicated squamation of *Guiyu*. The new squamation pattern (Fig. 5I), which divides the body of *Guiyu* into 16 areas, is more applicable to *Psarolepis*^5^, *Sparalepis*^24^ and other Silurian osteichthyans than the pattern designed by Esin^18^. The squamation pattern of *Andreolepis* designed by Chen *et al*.^6^ generally divides the antero-mid flank into three (dorsal, lateral and ventral) belts, but it lacks further partitions. The squamation pattern of 10 distinct scale areas in *Andreolepis* designed based on limited isolated scales. It may not contain the all morphotypes of scales of *Andreolepis*. In light of the new pattern, the squamation of *Andreolepis* may be more complicated than previously considered. We suggest that the anterior-mid lateral flank area, the dorsal flank area and the ventral flank area could be further subdivided into two areas respectively (Fig. 7).

The squamation of crown osteichthyans becomes more simplified regarding zonal differentiation (Fig. 7). *Heimenia ensis*, as a porolepiform sarcopterygian, has a relatively uniform squamation^51^. The same condition appears in primitive actinopterygians^18,23^. There are two hypotheses regarding the phylogenetic position of *Guiyu*. Our phylogenetic result resolves *Guiyu* as a stem sarcopterygian. Accordingly, the simplification of squamation in crown sarcopterygians and actinopterygians should result from the parallelism (Fig. 7, the cladogram on the left). If *Guiyu* is placed within stem osteichthyans as shown by some other phylogenetic works^27,28^, the simplification of squamation could be a synapomorphy of crown osteichthyans (Fig. 7, the cladogram on the right).

This squamation pattern of *Guiyu* has 3 merits. First, this pattern is established based on 5 articulated specimens of *Guiyu*, including the near-complete holotype, providing the less arguable evidence for the squamation evolution of early osteichthyans. Second, the new squamation pattern is imposed through the quantitative geometric morphometrics as well as clustering analysis and qualitative morphologic observations. Third, we divide the squamation into 4 main belts containing 16 areas and adopt an innovative nomenclature, making it more general and concise to describe the squamation of various fish taxa.

## Methods

### Materials

This study is based on specimens o*f Guiyu oneiros*, including the near-complete holotype (V15541a, b)^25^, one referred articulated specimen (V17914)^29^, three new articulated specimens (V25047, V25048, V25049.1) and six isolated scales, housed at the Institute of Vertebrate Paleontology and Paleoanthropology (IVPP), Chinese Academy of Sciences (CAS). The new articulated specimens of *Guiyu oneiros* were also collected from the muddy limestone of the Kuanti Formation (Late Ludlow, Silurian) in Qujing, Yunnan, China.

### CT scan and three-dimensional reconstruction

Three new specimens were prepared mechanically using pneumatic air scribes and needles under microscopes and scanned by the 225 kV high-resolution computed tomography (CT) apparatus (developed by Institute of High Energy Physics, Chinese Academy of Sciences) at the Key Laboratory of Vertebrate Evolution and Human Origins of Chinese Academy of Sciences. V25047 was scanned with beam energy of 150 kV and a flux of 100 μA at a resolution of 50.20 μm per pixel using a 360° rotation with a step size of 0.5°. A total of 720 projections were reconstructed in a 2,048*2,048 matrix of 1,536 slices using a two-dimensional reconstruction software developed by the Institute of High Energy Physics, CAS. V25048 was scanned with beam energy of 140 kV and a flux of 120 μA at a resolution of 39.21μm per pixel using a 360° rotation with a step size of 0.5°. A total of 720 projections were reconstructed in a 2,048*2,048 matrix of 1,536 slices using a two-dimensional reconstruction software developed by the Institute of High Energy Physics, CAS. V25049.1 was scanned with beam energy of 140 kV and a flux of 120 μA at a resolution of 28.23 μm per pixel using a 360° rotation with a step size of 0.5°. A total of 720 projections were reconstructed in a 2,048*2,048 matrix of 1,536 slices using a two-dimensional reconstruction software developed by the Institute of High Energy Physics, CAS. We reconstructed the CT models of scales by MIMICS (Materialise’s interactive medical image control system) 18.0.

### Geometric morphometric analysis

A cluster analysis was performed in the software of the Statistical Product and Service Solutions (SPASS) 24.0 and the CVA in Past^52^.

### Phylogenetic analysis

The character data entry and formatting were performed in Mesquite (version 2.5)^53^. The dataset was subject to the parsimony analysis in TNT software package^54^. The analyses were run using a traditional search strategy, with default settings apart from the following: 20,000 maximum trees in memory and 1,000 replications.

## Supplementary information

Supplementary Video 1

Supplementary Video 2

Supplementary Video 3

Supplementary Information

Dataset 1
