# Supplementary material for: Scale morphology and squamation pattern of Guiyu oneiros provide new insights into early osteichthyan body plan
Source: Sci Rep. 2019 Mar 13;9:4411. doi: 10.1038/s41598-019-40845-7 (PMC6416254; doi:10.1038/s41598-019-40845-7)
Supplement: Supplementary file 4 — Supplementary Information [file 41598_2019_40845_MOESM4_ESM.pdf]

Supplementary Information

Scale morphology and squamation pattern of *Guiyu oneiros* provide new insights into early osteichthyan body plan

Xindong Cui[1, 2, 3], Tuo Qiao[1, 2] & Min Zhu[1, 2, 3]

[1]Key Laboratory of Vertebrate Evolution and Human Origins of Chinese Academy of Sciences, Institute of Vertebrate Paleontology and Paleoanthropology, Chinese Academy of Sciences, Beijing 100044, China, [2]CAS Center for Excellence in Life and Paleoenvironment, Beijing, 100044, China, [3]University of Chinese Academy of Sciences, Beijing 100049, China

Corresponding author: Min Zhu, zhumin@ivpp.ac.cn

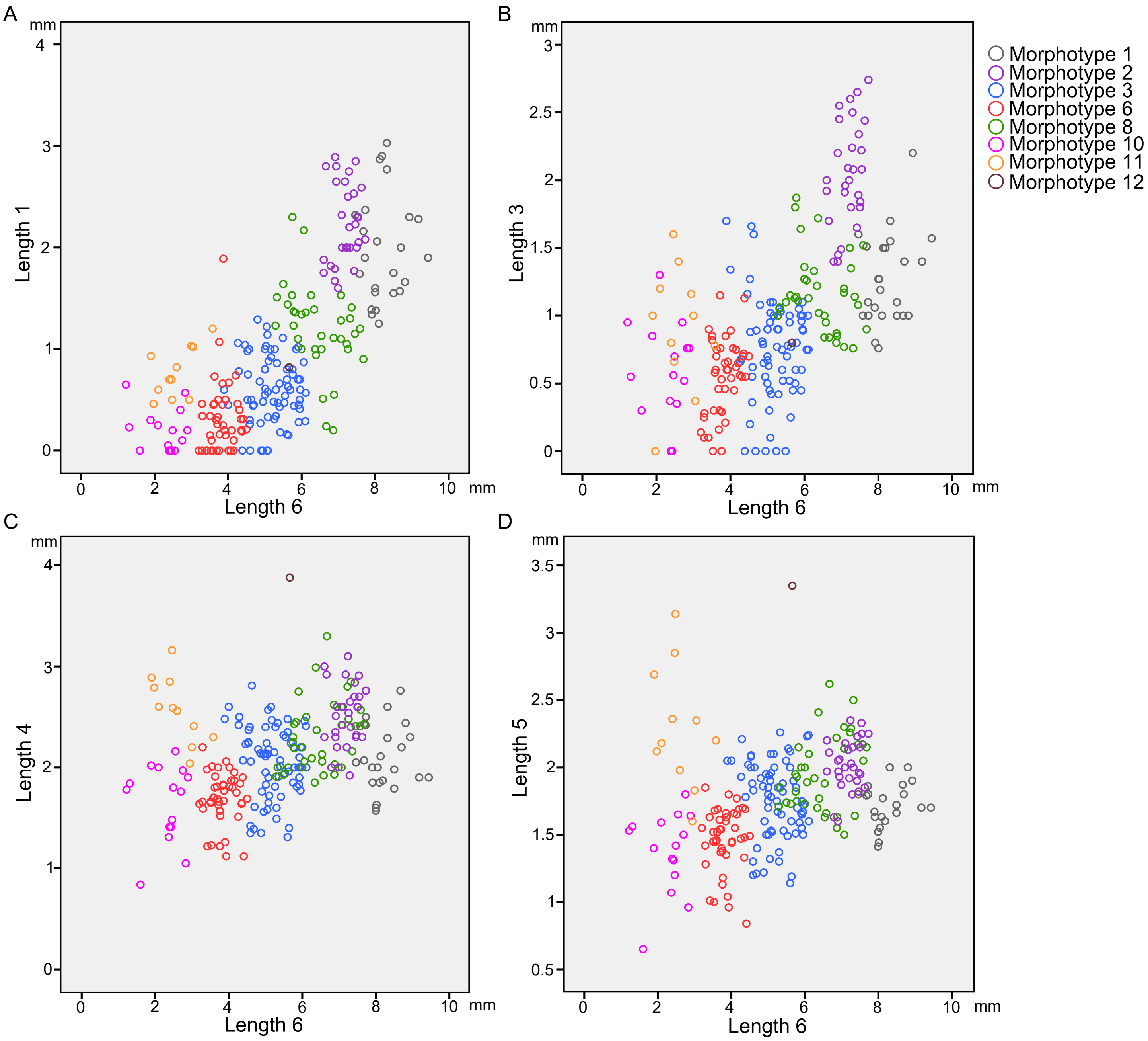

Supplementary Figure 1 | Scatter plots of the scales classified by cluster analysis. (A) Length 6 to Length 1. (B) Length 6 to Length 3. (C) Length 6 to Length 4. (D) Length 6 to Length 5.

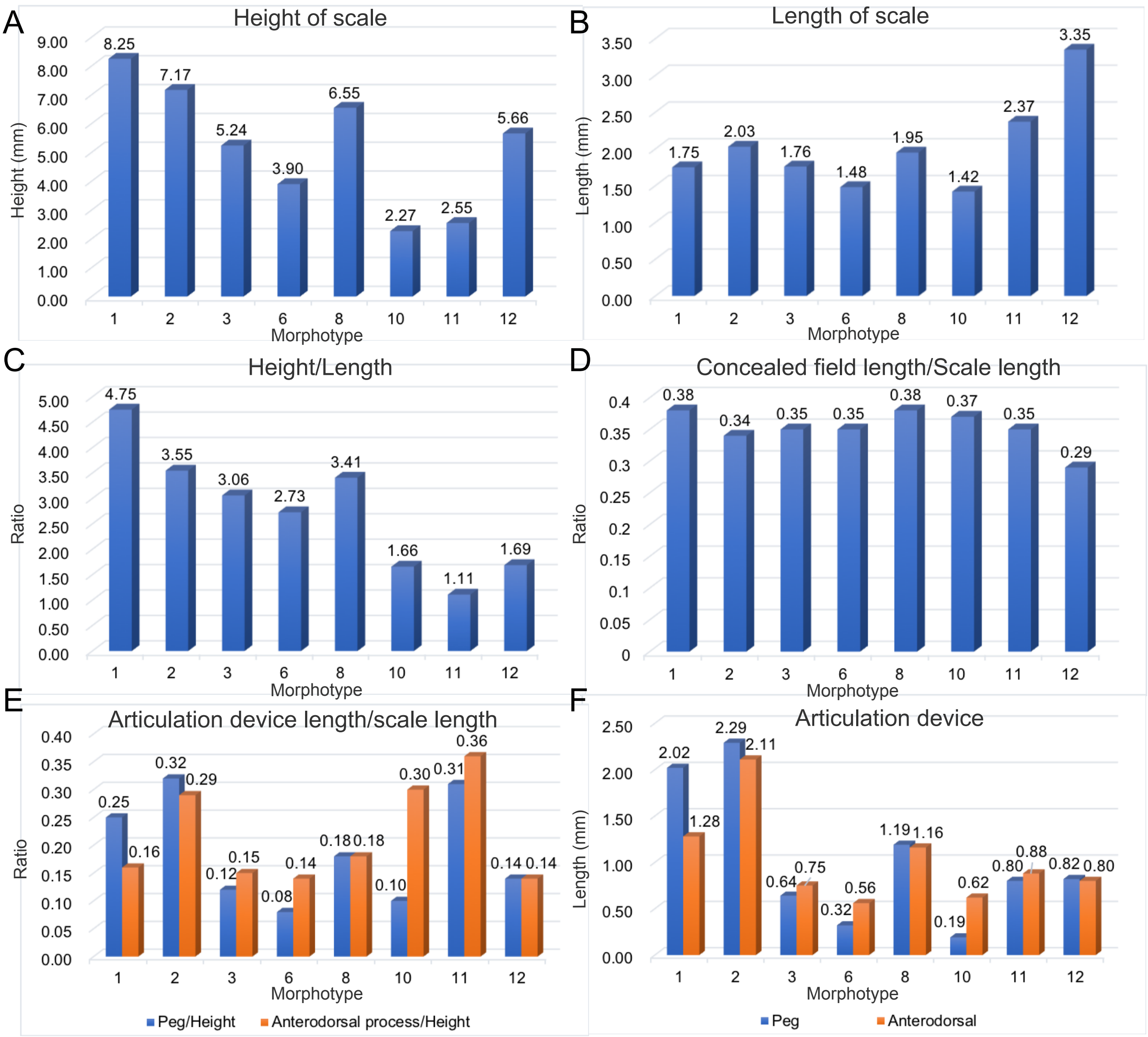

Supplementary Figure 2 | Bar graphs of the measurement of the scales classified by cluster analysis. (A) Height of scales. (B) Length of scales. (C) Ratio of height to length. (D) Ratio of concealed field length to scale length. (E) Ratio of articulation length to scale length. (F) Length of articulation devices.

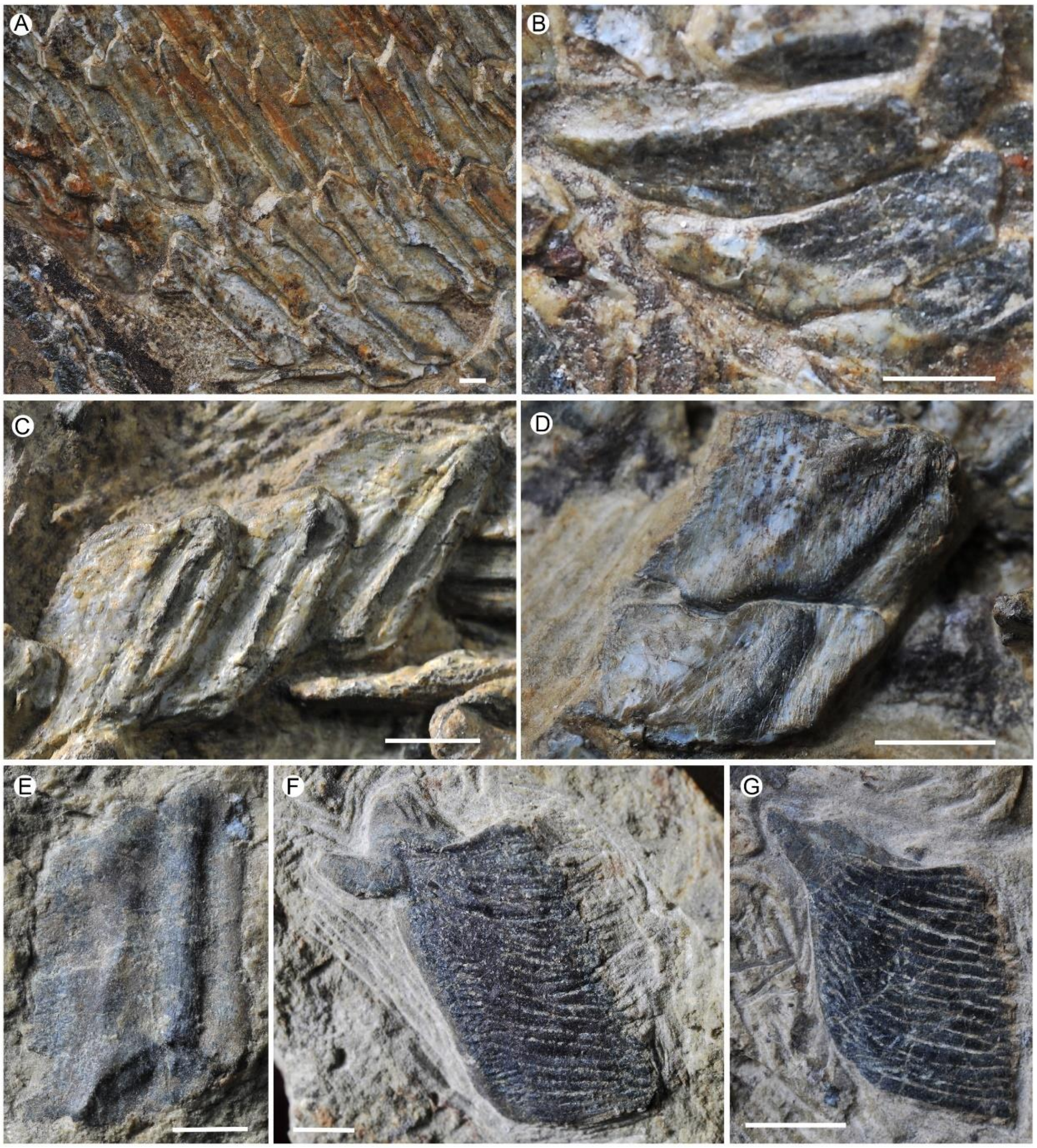

Supplementary Figure 3 | Scales of *Guiyu oneiros*. (A–D) V15541. Morphotype 2 (A), Morphotype 12 (B), Morphotype 3 (C), Morphotype 9 (D). (E) Morphotype 3, V25049.4. (F) Morphotype 2, V25049.5. (G) Morphotype 4, V25049.6. Scale bar = 2 mm.

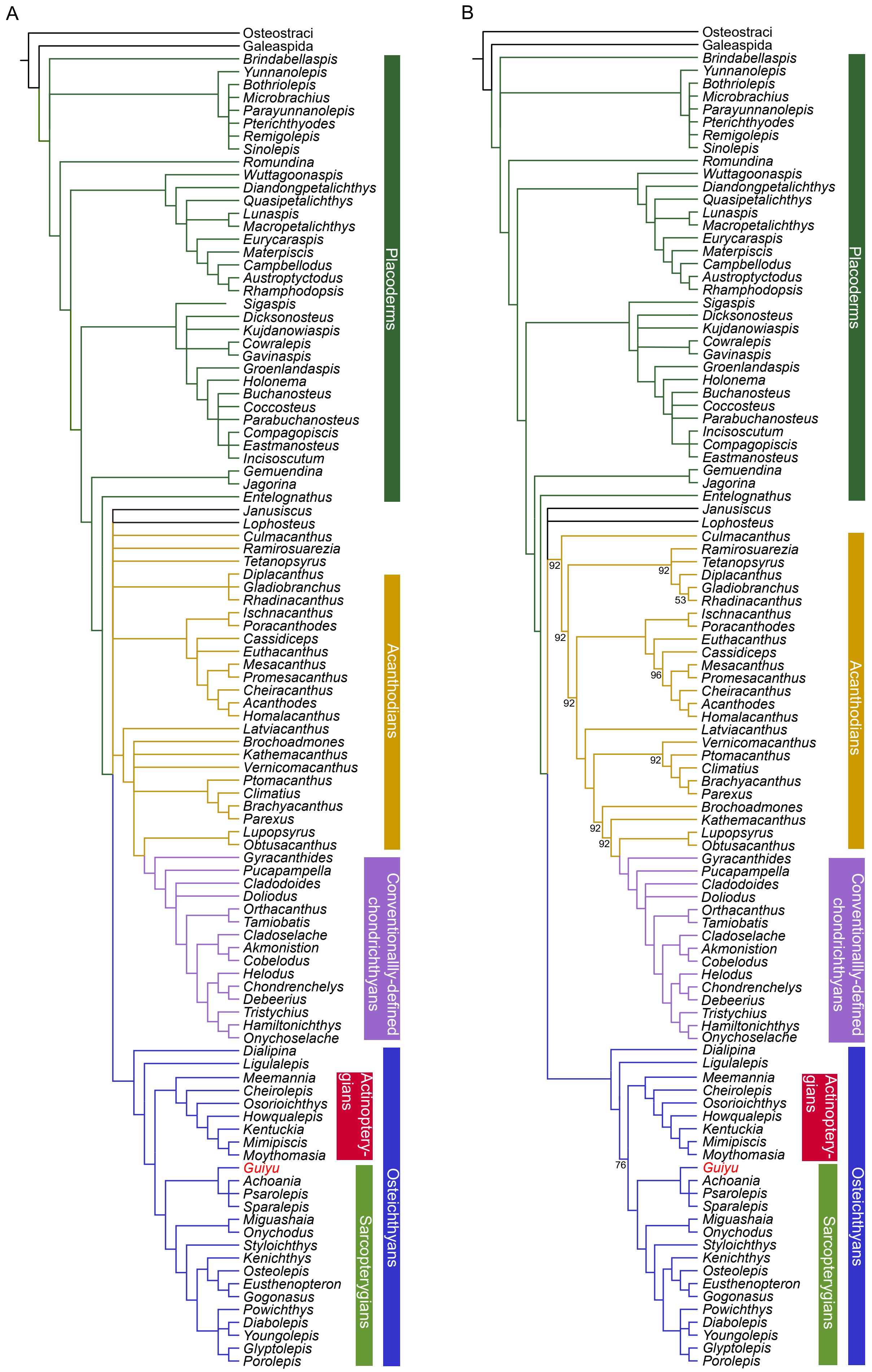

Supplementary Figure 4 | Phylogenetic relationships of gnathostomes. (A) The strict consensus tree of 1248 most parsimonious trees. (B)50% Majority-rule consensus tree. Numbers on branches indicate the percentage of most-parsimonious trees that contain a particular clade (100% unless otherwise indicated).

Supplementary Table 1 | Measurement of the scales of *Guiyu oneiros*.

**Number**	Group	Pre-Group	Morpho type	L1 (mm)	L3 (mm)	L4 (mm)	L5 (mm)	L6 (mm)	L7 (mm)	L6/L5	L7/L5	L1/L6	L3/L6
V25047-2-418	A	A	2	1.90	1.10	2.42	1.86	7.72	0.70	4.15	0.38	0.25	0.14
V25047-2-637	A	A	2	2.32	1.60	2.05	1.80	7.46	0.70	4.14	0.39	0.31	0.21
V25048-01	A	A	2	2.77	1.55	2.29	2.00	8.32	0.74	4.16	0.37	0.33	0.19
V25048-149	A	A	2	1.38	1.27	1.63	1.44	8.02	0.63	5.57	0.44	0.17	0.16
V25048-155	A	A	2	1.56	1.27	1.60	1.52	8.00	0.54	5.26	0.36	0.20	0.16
V25048-161	A	A	2	1.60	0.76	1.57	1.41	8.00	0.73	5.67	0.52	0.20	0.10
V25048-21	A	A	2	3.03	0.69	2.60	2.00	8.32	0.67	4.16	0.34	0.36	0.08
V25048-38	A	A	2	2.30	2.20	2.30	1.90	8.93	0.65	4.70	0.34	0.26	0.25
V25048-54	A	A	2	1.66	1.00	2.44	2.00	8.81	0.67	4.41	0.34	0.19	0.11
V25048-55	A	A	2	1.57	1.00	2.76	1.87	8.67	0.77	4.64	0.41	0.18	0.12
V25048-57	A	A	2	1.34	0.80	1.85	1.63	7.91	0.63	4.85	0.39	0.17	0.10
V25048-60	A	A	2	2.87	1.50	2.11	1.77	8.13	0.62	4.59	0.35	0.35	0.18
V25048-61	A	A	2	2.06	1.19	2.00	1.55	8.05	0.60	5.19	0.39	0.26	0.15
V25048-62	A	A	2	2.37	1.00	2.50	1.80	7.73	0.65	4.29	0.36	0.31	0.13
V25048-82	A	A	2	2.16	1.51	2.07	1.79	7.68	0.57	4.29	0.32	0.28	0.20
V25048-86	A	A	2	1.39	1.06	2.00	1.67	7.90	0.63	4.73	0.38	0.18	0.13
V25048-97	A	A	2	1.74	1.00	2.41	2.17	7.58	0.61	3.49	0.28	0.23	0.13
V25048-98	A	A	2	1.55	1.00	1.79	1.66	8.50	0.56	5.12	0.34	0.18	0.12
V25048-99	A	A	2	1.75	1.10	1.98	1.72	8.50	0.68	4.94	0.40	0.21	0.13
V25047-1-195	A	A	2	2.00	1.40	2.20	1.80	8.70	0.84	4.83	0.47	0.23	0.16
V25047-1-213	A	A	2	2.90	1.50	1.87	1.60	8.19	0.60	5.12	0.38	0.35	0.18
V25047-1-218	A	A	2	1.90	1.57	1.90	1.70	9.44	0.54	5.55	0.32	0.20	0.17
V25047-1-264	A	A	2	2.28	1.40	1.90	1.70	9.18	0.95	5.40	0.56	0.25	0.15
V25047-2-2	B	B	3	0.28	0.96	1.90	1.65	5.92	0.62	3.59	0.38	0.05	0.16
V25047-2-3	B	B	3	0.33	1.02	1.90	1.78	5.08	0.67	2.85	0.38	0.06	0.20
V25047-2-8	B	B	3	0.15	0.77	1.40	1.19	5.64	0.52	4.74	0.44	0.03	0.14
V25047-2-10	B	B	3	0.16	0.52	1.31	1.14	5.60	0.47	4.91	0.41	0.03	0.09
V25047-2-11	B	B	3	1.06	0.66	2.42	1.93	4.27	0.61	2.21	0.32	0.25	0.15
V25047-2-12	B	B	3	0.43	0.75	2.01	1.88	5.40	0.41	2.87	0.22	0.08	0.14
V25047-2-14	B	B	3	0.47	0.70	1.94	1.49	5.53	0.44	3.71	0.30	0.08	0.13
V25047-2-17	B	B	3	0.27	0.79	1.35	1.22	4.88	0.42	4.00	0.34	0.06	0.16
V25047-2-29	B	B	3	0.18	0.52	1.71	1.53	5.42	0.63	3.54	0.41	0.03	0.10
V25047-2-33	B	B	3	0.47	1.03	1.91	1.65	5.36	0.43	3.25	0.26	0.09	0.19
V25047-2-43	B	B	3	1.00	1.10	2.60	2.10	5.15	0.72	2.45	0.34	0.19	0.21
V25047-2-45	B	B	3	0.34	0.42	1.49	1.37	5.30	0.56	3.87	0.41	0.06	0.08
V25047-2-52	B	B	3	0.78	0.68	2.50	2.21	4.29	0.65	1.94	0.29	0.18	0.16
V25047-2-154	B	B	3	0.00	0.55	2.13	1.95	4.94	0.61	2.53	0.31	0.00	0.11
V25047-2-250	B	B	3	0.45	0.60	2.10	1.75	5.92	0.59	3.38	0.34	0.08	0.10

V25047-2-266	B	B	3	0.70	0.90	2.00	1.50	5.95	0.74	3.97	0.49	0.12	0.15
V25047-2-300	B	B	3	0.00	0.62	2.06	1.83	4.60	0.65	2.51	0.36	0.00	0.13
V25047-2-419	B	B	3	0.68	0.81	2.14	1.86	4.86	0.72	2.61	0.39	0.14	0.17
V25047-2-420	B	B	3	0.86	1.05	2.44	2.26	5.33	0.80	2.36	0.35	0.16	0.20
V25047-2-444	B	B	3	0.75	0.88	2.37	2.09	4.54	0.60	2.17	0.29	0.17	0.19
V25047-2-452	B	B	3	0.00	0.65	1.76	1.60	4.90	0.53	3.06	0.33	0.00	0.13
V25047-2-458	B	B	3	0.00	0.10	2.18	1.96	5.08	0.61	2.59	0.31	0.00	0.02
V25047-2-473	B	B	3	0.00	0.45	2.13	1.90	5.06	0.63	2.66	0.33	0.00	0.09
V25047-2-506	B	B	3	0.48	0.00	2.15	1.75	5.24	0.56	2.99	0.32	0.09	0.00
V25047-2-509	B	B	3	0.48	0.00	1.56	1.50	4.95	0.46	3.30	0.31	0.10	0.00
V25047-2-512	B	B	3	0.75	0.60	2.20	1.85	5.76	0.60	3.11	0.32	0.13	0.10
V25047-2-547	B	B	3	0.29	1.00	2.41	2.24	6.10	0.90	2.72	0.40	0.05	0.16
V25047-2-636	B	B	3	0.31	0.77	1.81	1.67	5.26	0.48	3.15	0.29	0.06	0.15
V25047-2-645	B	B	3	0.80	0.95	2.35	2.12	5.60	0.72	2.64	0.34	0.14	0.17
V25047-2-651	B	B	3	0.54	0.78	2.30	2.14	5.55	0.70	2.59	0.33	0.10	0.14
V25048-02	B	B	3	1.00	0.70	2.40	1.75	5.00	0.71	2.86	0.41	0.20	0.14
V25048-028	B	B	3	0.70	1.00	2.00	1.66	5.90	0.60	3.55	0.36	0.12	0.17
V25048-04	B	B	3	0.60	0.45	2.24	1.90	5.69	0.59	2.99	0.31	0.11	0.08
V25048-05	B	B	3	0.61	0.90	1.83	1.50	5.01	0.50	3.34	0.33	0.12	0.18
V25048-06	B	B	3	0.50	0.20	2.25	2.00	4.53	0.58	2.27	0.29	0.11	0.04
V25048-100	B	B	3	0.80	0.67	1.95	1.85	5.00	0.57	2.70	0.31	0.16	0.13
V25048-110	B	B	3	1.29	1.08	2.18	1.92	4.80	0.51	2.50	0.27	0.27	0.23
V25048-117	B	B	3	0.44	0.80	2.00	1.70	5.79	0.74	3.41	0.44	0.08	0.14
V25048-119	B	B	3	0.41	1.00	1.77	1.50	5.95	0.50	3.97	0.33	0.07	0.17
V25048-122	B	B	3	1.22	0.30	1.58	1.32	5.05	0.52	3.83	0.39	0.24	0.06
V25048-124	B	B	3	0.98	1.27	2.24	2.08	4.53	0.60	2.18	0.29	0.22	0.28
V25048-126	B	B	3	0.63	0.25	1.96	1.60	5.63	0.54	3.52	0.34	0.11	0.04
V25048-15	B	B	3	1.14	0.95	2.57	2.10	5.07	0.67	2.41	0.32	0.22	0.19
V25048-150	B	B	3	0.88	0.42	1.72	1.52	5.10	0.83	3.36	0.55	0.17	0.08
V25048-151	B	B	3	1.04	1.16	2.23	2.02	4.46	0.81	2.21	0.40	0.23	0.26
V25048-156	B	B	3	0.58	1.10	1.65	1.53	5.08	0.59	3.32	0.39	0.11	0.22
V25048-16	B	B	3	1.00	0.90	2.40	1.85	5.27	0.71	2.85	0.38	0.19	0.17
V25048-160	B	B	3	0.50	1.60	2.81	2.00	4.63	1.09	2.32	0.55	0.11	0.35
V25048-166	B	B	3	0.45	0.89	1.61	1.40	4.70	0.40	3.36	0.29	0.10	0.19
V25048-168	B	D	3	1.23	1.00	1.91	1.71	5.29	0.53	3.09	0.31	0.23	0.19
V25048-169	B	D	3	1.51	1.06	2.00	1.85	5.33	0.57	2.88	0.31	0.28	0.20
V25048-17	B	B	3	0.70	1.00	2.48	2.00	5.60	0.62	2.80	0.31	0.13	0.18
V25048-201	B	B	3	0.55	0.90	2.14	1.80	5.00	0.51	2.78	0.28	0.11	0.18
V25048-210	B	B	3	0.60	0.90	1.60	1.30	5.30	0.50	4.08	0.38	0.11	0.17
V25048-224	B	B	3	0.80	0.50	1.85	1.51	5.75	0.47	3.81	0.31	0.14	0.09
V25048-26	B	B	3	1.00	1.66	2.40	1.88	4.57	0.79	2.43	0.42	0.22	0.36
V25048-50	B	B	3	0.66	0.60	2.07	1.82	5.40	0.42	2.97	0.23	0.12	0.11

V25048-53	B	B	3	0.60	0.89	2.20	1.72	5.96	0.59	3.47	0.34	0.10	0.15
V25048-59	B	B	3	0.57	0.75	2.00	1.60	6.11	0.51	3.82	0.32	0.09	0.12
V25048-87	B	B	3	0.92	1.00	2.47	2.00	5.17	0.81	2.59	0.41	0.18	0.19
V25048-93	B	B	3	1.02	0.45	1.83	1.55	5.91	0.72	3.81	0.46	0.17	0.08
V2504961	B	B	3	1.11	0.00	2.32	2.05	5.49	0.64	2.68	0.31	0.20	0.00
V2504964	B	B	3	1.03	0.63	2.24	1.94	5.03	0.67	2.59	0.35	0.20	0.13
V25047-1-196	B	B	3	0.80	0.50	2.11	1.70	5.00	0.53	2.94	0.31	0.16	0.10
V25047-2-1	C	B	8	0.26	0.36	1.35	1.20	4.59	0.70	3.83	0.58	0.06	0.08
V25047-2-4	C	C	8	0.19	1.13	1.90	1.69	4.38	0.66	2.59	0.39	0.04	0.26
V25047-2-5	C	C	8	0.31	0.70	1.64	1.33	4.35	0.46	3.27	0.35	0.07	0.16
V25047-2-6	C	C	8	0.21	0.70	1.69	1.49	4.50	0.51	3.02	0.34	0.05	0.16
V25047-2-20	C	C	8	0.15	0.66	1.12	0.96	3.93	0.36	4.09	0.38	0.04	0.17
V25047-2-23	C	C	8	0.16	0.29	1.52	1.18	3.77	0.34	3.19	0.29	0.04	0.08
V25047-2-24	C	B	8	0.30	0.55	1.42	1.30	4.59	0.54	3.53	0.42	0.07	0.12
V25047-2-67	C	C	8	0.00	0.00	1.22	1.13	3.76	0.42	3.33	0.37	0.00	0.00
V25047-2-68	C	C	8	0.31	0.55	1.12	0.84	4.41	0.43	5.25	0.51	0.07	0.12
V25047-2-82	C	B	8	0.44	0.00	1.39	1.21	4.68	0.42	3.87	0.35	0.09	0.00
V25047-2-130	C	C	8	0.00	0.14	1.64	1.55	3.20	0.45	2.06	0.29	0.00	0.04
V25047-2-148	C	C	8	0.00	0.10	1.98	1.63	3.41	0.40	2.09	0.25	0.00	0.03
V25047-2-150	C	C	8	0.25	0.21	1.67	1.35	3.86	0.40	2.86	0.30	0.06	0.05
V25047-2-225	C	C	8	0.20	0.72	1.65	1.48	4.36	0.60	2.95	0.41	0.05	0.17
V25047-2-302	C	C	8	0.00	0.76	1.87	1.66	4.07	0.62	2.45	0.37	0.00	0.19
V25047-2-342	C	B	8	0.45	1.34	2.60	2.05	4.00	0.80	1.95	0.39	0.11	0.34
V25047-2-359	C	B	8	0.60	1.70	2.48	2.05	3.89	0.70	1.90	0.34	0.15	0.44
V25047-2-455	C	C	8	0.00	0.75	1.87	1.77	4.15	0.59	2.34	0.33	0.00	0.18
V25047-2-511	C	C	8	0.00	0.58	1.70	1.45	3.58	0.51	2.47	0.35	0.00	0.16
V25047-2-516	C	C	8	0.10	0.30	2.01	1.68	3.55	0.48	2.11	0.29	0.03	0.08
V25047-2-517	C	C	8	0.00	0.10	2.20	1.85	3.29	0.50	1.78	0.27	0.00	0.03
V25047-2-560	C	C	8	0.30	0.46	1.81	1.61	3.68	0.47	2.29	0.29	0.08	0.13
V25047-2-563	C	C	8	0.00	0.00	1.23	1.00	3.53	0.54	3.53	0.54	0.00	0.00
V25047-2-584	C	C	8	0.10	0.62	1.95	1.65	4.15	0.53	2.52	0.32	0.02	0.15
V25047-2-648	C	C	8	0.00	0.16	1.69	1.54	3.69	0.41	2.40	0.27	0.00	0.04
V25048-07	C	C	8	0.26	0.30	2.00	1.37	3.71	0.54	2.71	0.39	0.07	0.08
V25048-101	C	C	8	0.66	0.46	1.82	1.66	3.85	0.59	2.32	0.36	0.17	0.12
V25048-102	C	C	8	0.73	0.75	1.91	1.69	3.63	0.54	2.15	0.32	0.20	0.21
V25048-112	C	C	8	0.33	1.15	1.63	1.54	3.72	0.47	2.42	0.31	0.09	0.31
V25048-113	C	C	8	0.20	0.60	1.26	1.04	3.90	0.47	3.75	0.45	0.05	0.15
V25048-130	C	C	8	0.46	0.25	1.66	1.42	3.29	0.55	2.32	0.39	0.14	0.08
V25048-131	C	C	8	0.40	0.55	1.84	1.70	4.31	0.47	2.54	0.28	0.09	0.13
V25048-164	C	C	8	0.45	0.60	1.70	1.46	3.60	0.42	2.47	0.29	0.13	0.17
V25048-165	C	C	8	0.50	0.76	1.66	1.40	3.73	0.43	2.66	0.31	0.13	0.20
V25048-178	C	C	8	0.34	0.28	1.59	1.28	3.30	0.48	2.58	0.38	0.10	0.08

V25048-187	C	C	8	1.89	0.85	1.77	1.63	3.87	0.59	2.37	0.36	0.49	0.22
V25048-190	C	B	8	0.00	0.00	2.07	1.78	4.39	0.69	2.47	0.39	0.00	0.00
V25048-195	C	C	8	0.45	0.65	1.83	1.60	3.85	0.47	2.41	0.29	0.12	0.17
V25048-209	C	C	8	0.20	0.45	1.80	1.55	4.10	0.52	2.65	0.34	0.05	0.11
V25048-227	C	C	8	0.50	0.60	2.06	1.80	3.93	0.56	2.18	0.31	0.13	0.15
V25048-30	C	C	8	0.46	0.70	1.80	1.52	3.62	0.55	2.38	0.36	0.13	0.19
V25048-68	C	C	8	0.74	0.65	1.75	1.69	4.21	0.55	2.49	0.33	0.18	0.15
V25048-70	C	C	8	0.46	0.56	1.51	1.47	4.26	0.45	2.90	0.31	0.11	0.13
V25048-79	C	C	8	0.00	0.90	1.22	1.01	3.42	0.47	3.39	0.47	0.00	0.26
V25048-83	C	C	8	0.67	0.54	2.00	1.45	4.02	0.54	2.77	0.37	0.17	0.13
V25048-88	C	C	8	0.34	0.82	1.66	1.45	3.51	0.40	2.42	0.28	0.10	0.23
V25048-89	C	C	8	0.00	0.89	1.81	1.44	4.01	0.44	2.78	0.31	0.00	0.22
V25048-90	C	C	8	0.15	0.85	1.65	1.52	3.51	0.45	2.31	0.30	0.04	0.24
V2504907	C	C	8	1.07	0.53	1.57	1.38	3.76	0.47	2.72	0.34	0.28	0.14
V25047-2-44	D	D	4	1.23	1.87	2.43	1.75	5.78	0.62	3.30	0.35	0.21	0.32
V25047-2-73	D	D	4	0.55	0.85	2.03	1.63	6.88	0.64	4.22	0.39	0.08	0.12
V25047-2-173	D	D	4	1.00	1.72	2.99	2.41	6.37	0.90	2.64	0.37	0.16	0.27
V25047-2-234	D	D	4	1.10	1.64	2.75	2.00	5.90	0.65	2.95	0.33	0.19	0.28
V25047-2-249	D	E	4	1.79	1.40	2.35	2.05	6.90	0.65	3.37	0.32	0.26	0.20
V25047-2-287	D	D	4	1.28	1.17	2.42	2.14	7.07	0.90	3.30	0.42	0.18	0.17
V25047-2-308	D	D	4	0.24	0.84	3.30	2.62	6.67	0.82	2.55	0.31	0.04	0.13
V25047-2-354	D	D	4	1.00	1.35	2.48	2.29	7.26	0.72	3.17	0.31	0.14	0.19
V25047-2-365	D	D	4	0.20	0.80	2.62	2.22	6.86	0.70	3.09	0.32	0.03	0.12
V25047-2-382	D	B	4	0.87	0.75	2.46	2.23	6.06	0.64	2.72	0.29	0.14	0.12
V25047-2-390	D	D	4	0.90	0.90	2.43	2.15	7.68	0.73	3.57	0.34	0.12	0.12
V25047-2-401	D	D	4	1.20	1.52	2.57	2.05	7.59	0.73	3.70	0.36	0.16	0.20
V25047-2-640	D	D	4	1.05	1.50	2.80	2.26	7.23	0.83	3.20	0.37	0.15	0.21
V25047-2-647	D	E	4	1.60	1.49	2.00	1.87	6.99	0.61	3.74	0.33	0.23	0.21
V25048-108	D	D	4	1.37	1.14	2.19	1.93	5.78	0.81	2.99	0.42	0.24	0.20
V25048-120	D	D	4	1.13	1.00	2.06	1.63	6.54	0.61	4.01	0.37	0.17	0.15
V25048-121	D	E	4	1.82	1.40	2.00	1.63	6.79	0.60	4.17	0.37	0.27	0.21
V25048-128	D	D	4	1.53	1.33	2.09	1.92	6.26	0.76	3.26	0.40	0.24	0.21
V25048-143	D	D	4	1.64	1.10	1.93	1.74	5.50	0.60	3.16	0.34	0.30	0.20
V25048-144	D	D	4	1.44	1.15	2.00	1.73	5.62	0.64	3.25	0.37	0.26	0.20
V25048-146	D	D	4	1.34	1.36	2.00	1.73	6.00	0.59	3.47	0.34	0.22	0.23
V25048-147	D	D	4	1.39	1.22	1.85	1.70	6.34	0.63	3.73	0.37	0.22	0.19
V25048-152	D	D	4	0.51	0.95	1.92	1.66	6.58	0.61	3.96	0.37	0.08	0.14
V25048-161	D	D	4	2.17	1.26	2.06	1.89	6.06	0.74	3.21	0.39	0.36	0.21
V25048-48	D	D	4	1.36	1.11	2.45	2.15	5.83	0.63	2.71	0.29	0.23	0.19
V25048-49	D	D	4	1.36	1.13	2.50	2.10	6.11	0.60	2.91	0.29	0.22	0.18
V25048-56	D	D	4	1.10	0.77	2.00	1.50	7.07	0.78	4.71	0.52	0.16	0.11
V25048-67	D	D	4	0.94	1.02	2.37	1.77	6.39	0.80	3.61	0.45	0.15	0.16

V25048-69	D	D	4	1.00	0.84	2.13	1.88	6.54	0.60	3.48	0.32	0.15	0.13
V25048-85	D	B	4	1.00	1.05	1.81	1.59	5.85	0.68	3.68	0.43	0.17	0.18
V25048-92	D	D	4	1.11	0.87	1.93	1.55	6.87	0.65	4.43	0.42	0.16	0.13
V25048-94	D	D	4	1.41	1.14	2.16	1.64	7.36	0.65	4.49	0.40	0.19	0.15
V25048-95	D	D	4	1.53	1.13	2.25	1.95	5.74	0.73	2.94	0.37	0.27	0.20
V25048-96	D	D	4	1.15	1.08	2.41	2.17	7.45	0.65	3.43	0.30	0.15	0.14
V25047-1-197	D	D	4	1.30	0.76	2.85	2.50	7.32	1.34	2.93	0.54	0.18	0.10
V25047-1-220	D	D	4	1.53	1.20	2.60	2.30	7.07	1.20	3.07	0.52	0.22	0.17
V25047-1-34	D	D	4	1.00	1.27	2.20	2.00	6.00	1.18	3.00	0.59	0.17	0.21
V25047-2-48	E	E	1	2.32	1.91	2.42	2.00	7.09	0.69	3.55	0.35	0.33	0.27
V25047-2-49	E	E	1	2.50	1.80	2.54	2.03	7.26	0.67	3.58	0.33	0.34	0.25
V25047-2-50	E	E	1	2.59	2.44	2.30	1.85	7.63	0.68	4.12	0.37	0.34	0.32
V25047-2-277	E	E	1	2.00	1.80	2.60	2.15	7.50	0.64	3.49	0.30	0.27	0.24
V25047-2-278	E	E	1	1.67	2.20	2.30	1.60	6.90	0.68	4.31	0.43	0.24	0.32
V25047-2-318	E	E	1	1.88	2.00	3.00	2.20	6.60	0.72	3.00	0.33	0.28	0.30
V25047-2-400	E	E	1	2.65	2.09	2.92	2.13	7.18	0.77	3.37	0.36	0.37	0.29
V25047-2-411	E	E	1	2.23	1.89	2.30	1.82	7.46	0.51	4.10	0.28	0.30	0.25
V25047-2-412	E	E	1	2.08	2.74	2.76	2.25	7.73	0.68	3.44	0.30	0.27	0.35
V25047-2-414	E	E	1	2.20	2.50	2.65	2.15	7.30	0.68	3.40	0.32	0.30	0.34
V25047-2-416	E	E	1	2.30	2.22	2.91	2.33	7.54	0.89	3.24	0.38	0.31	0.29
V25047-2-424	E	E	1	2.65	2.55	2.60	2.06	6.94	0.61	3.37	0.30	0.38	0.37
V25047-2-425	E	E	1	1.77	2.65	2.84	2.23	7.43	0.62	3.33	0.28	0.24	0.36
V25047-2-426	E	E	1	2.00	2.60	3.10	2.35	7.24	0.82	3.08	0.35	0.28	0.36
V25047-2-427	E	E	1	2.00	1.96	2.60	2.18	7.10	0.74	3.26	0.34	0.28	0.28
V25047-2-428	E	E	1	1.75	1.92	2.30	1.97	6.60	0.80	3.35	0.41	0.27	0.29
V25047-2-431	E	E	1	2.05	2.08	2.70	2.25	7.55	0.78	3.36	0.35	0.27	0.28
V25047-2-549	E	E	1	2.00	2.00	2.20	1.92	7.21	0.57	3.76	0.30	0.28	0.28
V25047-2-551	E	E	1	2.00	2.08	2.40	2.00	7.32	0.56	3.66	0.28	0.27	0.28
V25047-2-642	E	E	1	2.80	2.45	2.20	1.93	6.94	0.43	3.60	0.22	0.40	0.35
V25047-2-643	E	E	1	2.85	2.34	2.32	1.96	7.47	0.43	3.81	0.22	0.38	0.31
V25047-2-644	E	E	1	2.80	1.70	2.92	2.11	6.66	0.63	3.16	0.30	0.42	0.26
V25047-2-654	E	E	1	2.75	2.24	1.92	1.80	7.29	0.48	4.05	0.27	0.38	0.31
V25048-37	E	D	1	2.30	1.80	2.30	1.82	5.75	0.78	3.16	0.43	0.40	0.31
V25048-63	E	E	1	2.89	1.45	2.51	1.97	6.91	0.65	3.51	0.33	0.42	0.21
V25047-1-144	E	E	1	2.30	1.84	2.60	1.95	7.51	0.91	3.85	0.47	0.31	0.25
V25047-1-145	E	E	1	2.53	1.65	2.70	1.90	7.42	1.10	3.91	0.58	0.34	0.22
V25047-2-7	F	F	5	0.00	0.35	2.16	1.65	2.55	0.56	1.55	0.34	0.00	0.14
V25047-2-93	F	F	5	0.25	1.30	2.00	1.59	2.09	0.50	1.31	0.31	0.12	0.62
V25047-2-286	F	F	5	0.30	0.85	2.02	1.40	1.89	0.50	1.35	0.36	0.16	0.45
V25047-2-554	F	F	5	0.00	0.00	1.41	1.32	2.39	0.44	1.81	0.33	0.00	0.00
V25047-2-606	F	F	5	0.00	0.00	1.41	1.31	2.43	0.56	1.85	0.43	0.00	0.00
V25048-111	F	G	5	0.50	1.16	2.04	1.60	2.94	0.48	1.84	0.30	0.17	0.39

V25048-171	F	F	5	0.20	0.76	1.90	1.64	2.89	0.48	1.76	0.29	0.07	0.26
V25048-173	F	F	5	0.10	0.52	1.97	1.80	2.75	0.55	1.53	0.31	0.04	0.19
V25048-205	F	F	5	0.40	0.95	1.76	1.50	2.70	0.67	1.80	0.45	0.15	0.35
V25048-84	F	F	5	0.00	0.56	1.48	1.20	2.46	0.38	2.05	0.32	0.00	0.23
V2504908	F	F	5	0.23	0.55	1.84	1.56	1.31	0.91	0.84	0.58	0.18	0.42
V2504912	F	F	5	0.20	0.70	1.80	1.42	2.49	0.68	1.75	0.48	0.08	0.28
V2504952	F	F	5	0.05	0.37	1.31	1.07	2.37	0.34	2.21	0.32	0.02	0.16
V2504970	F	F	5	0.65	0.95	1.78	1.53	1.22	0.58	0.80	0.38	0.53	0.78
V2504983	F	F	5	0.00	0.30	0.84	0.65	1.60	0.21	2.46	0.32	0.00	0.19
V25047-2-88	G	G	6	1.20	0.78	2.30	2.20	3.58	0.76	1.63	0.35	0.34	0.22
V25047-2-89	G	G	6	0.93	1.00	2.89	2.69	1.90	0.90	0.71	0.33	0.49	0.53
V25047-2-94	G	G	6	0.60	1.20	2.60	2.18	2.10	0.69	0.96	0.32	0.29	0.57
V25047-2-95	G	G	6	0.82	1.40	2.56	1.98	2.60	0.72	1.31	0.36	0.32	0.54
V25047-2-280	G	G	6	0.46	0.00	2.79	2.12	1.97	0.69	0.93	0.33	0.23	0.00
V25047-2-311	G	G	6	0.70	1.60	3.16	2.85	2.46	0.90	0.86	0.32	0.28	0.65
V25047-2-314	G	G	6	0.50	0.66	2.59	3.14	2.48	0.90	0.79	0.29	0.20	0.27
V25047-2-315	G	G	6	1.02	0.37	2.41	2.35	3.05	0.86	1.30	0.37	0.33	0.12
V25047-2-343	G	G	6	1.03	1.00	2.20	1.83	3.00	0.57	1.64	0.31	0.34	0.33
V25047-1-41	G	G	6	0.70	0.80	2.85	2.36	2.40	1.28	1.02	0.54	0.29	0.33
V25047-2-309	H	H	7	0.82	0.80	3.88	3.35	5.66	0.96	1.69	0.29	0.14	0.14

L1: the length of the peg; L3: the length of the anterodorsal process; L4: the length of scale in the longitudinal body axis; L5: the length of the scale; L6: the length of the posterior margin of crown (or the height of the scale); L7: the length of the concealed field.

Supplementary Note 1: Taxa used in phylogenetic analysis with principal sources of data.

Outgroup (2 OTUs)

Osteostraci: Janvier 1981, 1985; Janvier et al. 2004;

Galeaspida: Halstead 1979; Janvier 1981; Wang 1991; Pan 1992; Gai et al. 2011;

Ingroup (102 OTUs)

Yunnanolepis: Zhang 1980; Zhu 1996; Giles et al. 2013;

Parayunnanolepis: Zhang et al. 2001; Zhu et al. 2012b;

Sinolepis: Liu and P'an 1958; Ritchie et al. 1992;

Microbrachius: Hemmings 1978; Long et al. 2015;

Bothriolepis: Young 1984; Janvier 1996; Arsenault et al. 2004; Downs and Donoghue 2009;

Pterichthyodes: Hemmings 1978;

Remigolepis: Denison 1978; Pan et al. 1980, 1987; Johanson 1997;

Diandongpetalichthys: P'an and Wang 1978; Zhu 1991;

Quasipetalichthys: Liu 1973;

Quasipetalichthys: Liu 1991;

Lunaspis: Heintz 1937; Gross 1961;

Macropetalichthys: Stensiö 1925, 1969; Gross 1935; Denison 1978; Young 1978;

Wuttagoonaspis: Ritchie 1973; Young and Goujet 2003;

Groenlandaspis: Ritchie 1975; Anderson et al. 1994;

Gavinaspis: Dupret and Zhu 2008;

Cowralepis: Ritchie 2005; Carr et al. 2009; Long et al. 2009;

Sigaspis: Goujet 1973;

Dicksonosteus: Goujet 1975, 1984;

Kujdanowiaspis: Stensiö 1969; Dupret 2010;

Buchanosteus: Long et al. 2014;

Parabuchanosteus: White and Toombs 1972; White 1978; Young 1979; Long, et al. 2014;

Holonema: Miles 1971; Denison 1978; Trinajstic et al. 2015;

Coccosteus: Gross 1935; Stensiö 1963; Miles and Westoll 1968;

Incisoscutum: Dennis and Miles 1981; Johanson and Smith 2005; Long, et al. 2009; Giles, et al. 2013;

Eastmanosteus: Dennis-Bryan 1987;

Compagopiscis: Gardiner and Miles 1994; Trinajstic, et al. 2015;

Materpiscis: Long et al. 2008;

Austroptyctodus: Miles and Young 1977; Long 1997;

Campbellodus: Miles and Young 1977; Long 1997;

Rhamphodopsis: Watson 1938; Miles 1967; Miles and Young 1977; Long 1997;

Brindabellaspis: Young 1980, 1986; Burrow and Turner 1998, 1999; Goujet and Young 2004;

Romundina: Ørvig 1975; Dupret et al. 2014;

Jagorina: Stensiö 1969;

Gemuendina: Gross 1963; Young 1986;

Entelognathus: Zhu et al. 2013;

Janusiscus: Giles et al. 2015;

Ramirosuarezia: Pradel et al. 2009;

Acanthodes: Miles 1968, 1973a, b; Jarvik 1977; Denison 1979; Coates and Davis 2010; Davis et al. 2012; Brazeau and de Winter 2015;

Brachyacanthus: Watson 1937; Denison 1979;

Brochoadmones: Bernacsek and Dineley 1977; Gagnier and Wilson 1996b; Hanke and Wilson 2006;

Cassidiceps: Gagnier and Wilson 1996a;

Cheiracanthus: Watson 1937; Miles 1973a; Denison 1979;

Climatius: Watson 1937; Ørvig 1967b; Miles 1973a, b;

Culmacanthus: Long 1983; Young 1989; Burrow and Young 2012;

Diplacanthus: Watson 1937; Miles 1973b; Denison 1979;

Euthacanthus: Watson 1937; Miles 1973a; Newman et al. 2011;

Gladiobranchus: Bernacsek and Dineley 1977; Hanke and Davis 2008;

Gyracanthides: Miles 1973a; Warren et al. 2000; Turner et al. 2005;

Homalacanthus: Watson 1937; Gagnier 1996;

Ischnacanthus: Watson 1937; Miles 1973a; Hermus 2003;

Kathemacanthus: Gagnier and Wilson 1996a; Hanke and Wilson 2010;

Latviacanthus: Schultze and Zidek 1982;

Lupopsyrus: Bernacsek and Dineley 1977; Hanke and Davis 2012;

Mesacanthus: Watson 1937; Miles 1973a;

Obtusacanthus: Hanke and Wilson 2004;

Parexus: Watson 1937; Miles 1973a;

Poracanthodes: Denison 1979; Valiukevicius 1992;

Promesacanthus: Hanke 2008;

Ptomacanthus: Miles 1973a, b; Brazeau 2009;

Rhadinacanthus: Traquair 1888; Miles 1973a;

Tetanopsyrus: Gagnier 1995; Gagnier et al. 1999; Hanke et al. 2001;

Vernicomacanthus: Miles 1973a;

Akmonistion: Coates and Sequeira 1998, 2001a, b; Coates et al. 1998;

Chondrenchelys: Moy-Thomas 1935;

Cladodoides: Gross 1937, 1938; Maisey 2005;

Cladoselache: Woodward and White 1938; Bendix- Almgreen 1975; Schaeffer 1981; Maisey 1989b, 2007;

Cobelodus: Zangerl and Case 1976; Maisey 2007;

Debeerius: Grogan and Lund 2000;

Doliodus: Miller et al. 2003; Maisey et al. 2009;

Hamiltonichthys: Maisey 1989a;

Helodus: Moy-Thomas J. A. 1936;

Onychoselache: Dick and Maisey 1980; Maisey 1980; Coates and Gess 2007;

Orthacanthus: Heidtke 1982, 1998; Soler-Gijón 1999;

Pucapampella: Maisey 2001;

Tamiobatis: Romer 1964; Schaeffer 1981; Williams 1998;

Tristychius: Woodward 1924; Coates and Gess 2007;

Lophosteus: Gross 1969, 1971; Otto 1991; Burrow 1995; Schultze and Märss 2004; Botella et al. 2007;

Dialipina: Schultze 1968, 1992; Schultze and Cumbaa 2001;

Ligulalepis: Schultze 1968; Burrow 1994; Basden et al. 2000; Basden and Young 2001;

Cheirolepis: Ørvig 1967a; Pearson and Westoll 1979; Pearson 1982; Arratia and Cloutier 1996, 2004;

Howqualepis: Long 1988;

Mimipiscis: Gardiner 1984; Choo 2011;

Moythomasia: Gardiner 1984;

Kentuckia: Rayner 1951;

Osorioichthys: Taverne 1997;

Meemannia: Zhu et al. 2006, 2010; Lu et al. 2016

Guiyu: Zhu et al. 2009, 2012a; Qiao and Zhu 2010;

Psarolepis: Zhu and Schultze 1997, 2001; Yu 1998; Zhu et al. 1999; Zhu and Yu 2004, 2009; Qu et al. 2010, 2013, 2015;

Sparalepis: Choo et al. 2017;

Achoania: Zhu et al. 2001; Zhu and Yu 2004, 2009;

Onychodus: Jessen 1966; Long 2001; Andrews et al. 2005;

Miguashaia: Schultze 1973; Cloutier 1996; Forey 1998; Forey et al. 2000;

Styloichthys: Zhu and Yu 2002, 2004;

Diabolepis: Chang and Yu 1984; Chang 1995;

Youngolepis: Chang and Yu 1981; Chang 1982, 1991, 2004; Chang and Smith 1992;

Powichthys: Jessen 1975, 1980; Clément and Janvier 2004; Clément and Ahlberg 2010;

Porolepis: Jarvik 1972, 1980; Clément 2004;

Glyptolepis: Andrews and Westoll 1970; Jarvik 1972; Ahlberg 1989; Cloutier and Ahlberg 1996;

Kenichthys: Chang and Zhu 1993; Zhu and Ahlberg 2004;

Osteolepis: Jarvik 1948,1980;

Gogonasus: Long 1985; Long et al. 1997, 2006; Holland and Long 2009;

Eusthenopteron: Jarvik 1980.

Supplementary Note 2: List of characters used in the phylogenetic analysis.

Characters 1-336 are from Choo et al. (2017), Characters 337-341 are from Lu et al. (2017), Characters 342-346 are newly added.

1. Tessellate prismatic calcified cartilage

    0 absent

    1 present

2. Perichondral bone

    0 present

    1 absent

3. Extensive endochondral ossification

    0 absent

    1 present

4. Dentine

    0 absent

    1 present

5. Dentine kind

    0 mesodentine

    1 semidentine

    2 orthodentine

6. Lepidotrichia or lepidotrichia-like scale alignment

    0 present

    1 absent

7. Body scale growth pattern

    0 monodontode

    1 polyodontode

8. Body scale growth concentric

    0 absent

    1 present

9. Body scales with peg-and-socket articulation

    0 absent

1 present

10. Body scale profile

0 distinct crown and base demarcated by a constriction (neck)

1 flattened

11. Body scales with bulging base

0 absent

1 present

12. Body scales with flattened base

0 present

1 absent

13. Flank scales alignment

0 vertical rows

1 oblique rows or hexagonal/rhombic packing

2 disorganised

14. Sensory line canal

0 passes between or beneath scales

1 passes over scales and/or is partially enclosed or surrounded by scales

2 perforates and passes through scales

15. Sensory line network

0 preserved as open grooves

1 pass through canals enclosed within dermal bones

16. Jugal portion of infraorbital canal joins supramaxillary canal

0 present

1 absent

17. Dermal skull roof

0 includes large dermal plates

1 consists of undifferentiated plates or tesserae

18. Tesserae morphology

0 large interlocking polygonal plates

1 microsquamose, not larger than body tesserae

19. Extent of dermatocranial cover

0 complete

1 incomplete (scale-free and elsewhere)

20. Endolymphatic ducts open in dermal skull roof

0 present

1 absent

21. Endolymphatic ducts with oblique course through dermal skull bones

0 absent

1 present

22. Series of paired median skull roofing bones that meet at the dorsal midline of the skull (rectilinear skull roof pattern)

0 absent

1 present

23. Consolidated cheek plates

0 absent

1 present

24. Pineal opening perforation in dermal skull roof

0 present

1 absent

25. Enlarged postorbital tessera separate from orbital series

0 absent

1 present

26. Bony hyoidean gill-cover series (branchiostegals)

0 absent

1 present

27. Branchiostegal plate series along ventral margin of lower jaw

0 absent

1 present

28. Branchiostegal ossifications

0 plate-like

1 narrow and ribbon-like

29. Branchiostegal ossifications

0 ornamented

1 unornamented

30. Imbricated branchiostegal ossifications

0 absent

1 present

31. Opercular cover of branchial chamber

0 complete or partial

1 separate gill covers and gill slits

32. Opercular (submarginal) ossification

0 absent

1 present

33. Shape of opercular (submarginal) ossification

0 broad plate that tapers towards its proximal end

1 narrow, rod-shaped

34. Gular plates

0 absent

1 present

35. Size of lateral gular plates

0 extending most of length of the lower jaw

1 restricted to theanterior third of the jaw (no longer than the width of three or four branchiostegals

36. Basihyal

0 present

1 absent, hyoid arch articulates directly with basibranchial

37. Interhyal

0 absent

1 present

38. Oral dermal tubercles borne on jaw cartilages

0 absent

1 present

39. Tooth whorls

0 absent

1 present

40. Bases of tooth whorls

0 single, continuous plate

1 some or all whorls consist of separate tooth units

41. Enlarged adsymphysial tooth whorl

0 absent

1 present

42. Teeth ankylosed to dermal bones

0 absent

1 present

43. Dermal jaw plates on biting surface of jaw cartilages

0 absent

1 present

44. Maxillary and dentary tooth-bearing bones

0 absent

1 present

45. Large otic process of the palatoquadrate

0 absent

1 present

46. Insertion area for jaw adductor muscles on palatoquadrate

0 ventral

1 lateral

47. Oblique ridge or groove along medial face of palatoquadrate

0 absent

1 present

48. Fenestration of palatoquadrate at basipterygoid articulation

0 absent

1 present

49. Perforate or fenestrate anterodorsal (metapterygoid) portion of palatoquadrate

0 absent

1 present

50. Pronounced dorsal process on Meckelian bone or cartilage

0 absent

1 present

51. Preglenoid process

0 absent

1 present

52. Jaw articulation located on rearmost extremity of mandible

0 absent

1 present

53. Precerebral fontanelle

0 absent

1 present

54. Median dermal bone of palate (parasphenoid)

0 absent

1 present

55. Nasal opening(s)

0 dorsal, placed between orbits

1 ventral and anterior to orbits

56. Olfactory tracts

0 short, with olfactory capsules situated close to telencephalon cavity

1 elongate and tubular (much longer than wide)

57. Prominent pre-orbital rostral expansion of the neurocranium

0 present

1 absent

58. Pronounced sub-ethmoidal keel

0 absent

1 present

59. Position of myodome for superior oblique eye muscles

0 posterior and dorsal to foramen for nerve II

1 anterior and dorsal to foramen

60. Endoskeletal cranial joint

0 absent

1 present

61. Spiracular groove on basicranial surface

0 absent

1 present

62. Spiracular groove on lateral commissure

0 absent

1 present

63. Subpituitary fenestra

0 absent

1 present

64. Supraorbital shelf broad with convex lateral margin

0 absent

1 present

65. Orbit dorsal or facing dorsolaterally, surrounded laterally by endocranium

0 present

1 absent

66. Extended prehypophysial portion of sphenoid

0 absent

1 present

67. Narrow interorbital septum

0 absent

1 present

68. Main trunk of facial nerve (N. VII)

0 is elongate and passes anterolaterally through orbital floor

1 is stout and divides within otic capsule at the level of the postorbital process

69. Hyoid ramus of facial nerve (N. VII) exits through posterior jugular opening

0 absent

1 present

70. Glossopharyngeal nerve (N. IX) exit

0 foramen situated posteroventral to otic capsule and anterior to metotic fissure

1 through metotic fissure

71. Short otico-occipital region of braincase

0 absent

1 present

72. Ethmoid region elongate with dorsoventrally deep lateral walls

0 absent

1 present

73. Basicranial morphology

0 platybasic

1 tropibasic

74. Ascending basisphenoid pillar pierced by common internal carotid

0 absent

1 present

75. Canal for efferent pseudobranchial artery within basicranial cartilage

0 absent

1 present

76. Position of hyomandibula articulation on neurocranium

0 absent

1 present

77. Canal for lateral dorsal aorta within basicranial cartilage

0 absent

1 present

78. Entrance of internal carotids

0 through separate openings flanking the hypophyseal opening or recess

1 through a common opening at the central midline of the basicranium

79. Position of basal/basipterygoid articulation

0 same anteroposterior level as hypophysial opening

1 anterior to hypophysial opening

80. Postorbital process articulates with palatoquadrate

0 absent

1 present

81. Labyrinth cavity

0 separated from the main neurocranial cavity by a cartilaginous or ossified capsular wall

1 skeletal capsular wall absent

82. Basipterygoid process (basal articulation) with vertically oriented component

0 absent

1 present

83. Pituitary vein canal

0 dorsal to level of basipterygoid process

1 flanked posteriorly by basipterygoid process

84. External (horizontal) semicircular canal

0 absent

1 present

85. Sinus superior

0 absent or indistinguishable from union of anterior and posterior canals with saccular chamber

1 present

86. External (horizontal) semicircular canal

0 joins the vestibular region dorsal to posterior ampulla

1 joins level with posterior ampulla

87. Trigemino-facial recess

0 absent

1 present

88. Posterior dorsal fontanelle

0 absent

1 present

89. Shape of posterior dorsal fontanelle

0 approximately as long as broad

1 much longer than wide, slot-shaped

90. Dorsal ridge

0 absent

1 present

91. Endolymphatic ducts

0 posteriodorsally angled tubes

1 tubes oriented vertically through median endolymphatic fossa

92. Ventral cranial fissure

0 absent

1 present

93. Metotic (otic-occipital) fissure

0 absent

1 present

94. Vestibular fontanelle

0 absent

1 present

95. Occipital arch wedged in between otic capsules

0 absent

1 present

96. Spino-occipital nerve foramina

0 two or more, aligned horizontally

1 one or two, dorsoventrally offset

97. Ventral notch between parachordals

0 absent

1 present or entirely unfused

98. Parachordal shape

0 broad, flat

1 keeled with sloping lateral margins

99. Hypotic lamina (and dorsally directed glossopharyngeal canal)

0 absent

1 present

100. Macromeric dermal shoulder girdle

0 present

1 absent

101. Dermal shoulder girdle composition

0 ventral and dorsal (scapular) components

1 ventral components only

102. Dermal shoulder girdle forming a complete ring around the trunk

0 present

1 absent

103. Pectoral fenestra completely encircled by dermal shoulder armour

0 present

1 absent

104. Median dorsal plate

0 absent

1 present

105. Pronounced internal crista (keel) on median dorsal surface of shoulder girdle

0 absent

1 present

106. Scapular process of shoulder endoskeleton

0 absent

1 present

107. Ventral margin of separate scapular ossification

0 horizontal

1 deeply angled

108. Cross sectional shape of scapular process

0 flattened or strongly ovate

1 subcircular

109. Flange on trailing edge of scapulocoracoid

0 absent

1 present

110. Scapular process with posterodorsal angle

0 absent

1 present

111. Endoskeletal postbranchial lamina on scapular process

0 present

1 absent

112. Mineralisation of internal surface of scapular blade

0 mineralised all around

1 unmineralised on internal face forming a hemicylindrical cross-section

113. Coracoid process

0 absent

1 present

114. Procoracoid mineralisation

0 absent

1 present

115. Fin base articulation on scapulocoracoid

0 stenobasal

1 eurybasal

116. Perforate propterygium

0 absent

1 present

117. Pelvic fins

0 absent

1 present

118. Intromittent organ containing bone, not associated with pelvic fins

0 absent

1 present

119. Dermal pelvic clasper ossifications

0 absent

1 present

120. Pectoral fins covered in macromeric dermal armour

0 absent

1 present

121. Pectoral fin base has large, hemispherical dermal component

0 absent

1 present

122. Dorsal fin spines

0 absent

1 present

123. Anal fin spine

0 absent

1 present

124. Paired fin spines

0 absent

1 present

125. Median fin spine insertion

0 shallow, not greatly deeper than dermal bones / scales

1 deep

126. Intermediate fin spines

0 absent

1 present

127. Prepectoral fin spines

0 absent

1 present

128. Fin spines with ridges

0 absent

1 present

129. Fin spines with nodes

0 absent

1 present

130. Fin spines with rows of large retrorse denticles

0 absent

1 present

131. Synarcual

0 absent

1 present

132. Number of dorsal fins, if present

0 one

1 two

133. Anal fin

0 absent

1 present

134. Caudal radials

0 extend beyond level of body wall and deep into hypochordal lobe

1 restricted to axial lobe

135. Median gular

0 present

1 absent

136. Acrodin

0 absent

1 present

137. Plicidentine

0 absent

1 simple or generalized polyplacodont

138. Peg on rhomboid scale

0 narrow

1 broad

139. Anterodorsal process on scale

0 absent

1 present

140. Epichordal lepidotrichia in caudal fin

0 absent

1 present

141. Dermal intracranial joint

0 absent

1 present

142. Posterior nostril

0 associated with orbit

1 not associated with orbit

143. Number of marginal bones alongside paired median skull roofing bones over the otico-occipital division of braincase

0 single

1 two or more

144. Type of dermal neck-joint

0 sliding, dermal shoulder girdle plate with flat articular flange

1 ginglymoid, dermal shoulder girdle plate with articular condyle

2 reversed ginglymoid, dermal shoulder girdle plate with articular fossa

3 spoon-like

4 simple contact

145. Posterior expansion of maxilla (maxilla cleaver-shaped)

0 present

1 absent

146. Contribution by maxilla to posterior margin of cheek

0 present

1 absent

147. Number of coronoids

0 more than three

1 three

148. Fangs of coronoids (sensu stricto)

0 absent

1 present

149. Eye stalk or unfinished area on neurocranial wall for eye stalk

0 absent

1 present

150. Articulation facet with hyomandibular

0 single-headed

1 double-headed

151. Basicranial fenestra

0 absent

1 present

152. Lateral cranial canal

0 absent

1 present

153. Midline canal in basicranium for dorsal aorta

0 absent

1 present

154. Ascending process of parasphenoid

0 absent

1 present

155. Shape of parasphenoid denticulated field

0 broad rhomboid or lozenge-shaped

1 broad, splint-shaped

2 slender, splint-shaped

156. Parasphenoid denticulated field with multifid anterior margin

0 absent

1 present

157. Resorption and redeposition of odontodes

0 lacking or partially developed

1 developed

158. Rostral tubuli

0 absent

1 present

159. Large unpaired median skull roofing bone anterior to the level of nasal capsules

0 absent

1 present

160. Number of nasals

0 many

1 one or two

161. Mesial margin of nasal

0 not notched

1 notched

162. Dermintermedial process

0 absent

1 present

163. Position of posterior nostril

0 external, far from jaw margin

1 external, close to jaw margin

164. Supraorbital (sensu Cloutier and Ahlberg 1996, including posterior tectal of Jarvik)

0 absent

1 present

165. Supraorbital, preorbital and nasal

0 unfused

1 fused

166. Tectal (sensu Cloutier and Ahlberg 1996, not counting the posterior tectal of Jarvik)

0 absent

1 present

167. Lateral plate

0 absent

1 present

168. Location of pineal foramen/eminence

0 level with posterior margin of orbits

1 well posterior of orbits

169. Parietals (preorbitals of placoderms) surround pineal foramen or eminence

0 yes

1 no

170. Complete enclosure of spiracle by skull roof bones

0 absent

1 present

171. paranuchal number

0 one pair

1 two pairs

172. Large unpaired median bone contributing to posterior margin of skull roof

0 absent

1 present

173. Contact of nuchal or centronuchal plate with paired preorbital plates

0 absent

1 present

174. Posterior process of the paranuchal plate behind the nuchal plate (dorsal face)

0 absent

1 present

175. Junction of posterior pitline and main lateral line

0 far in front of posterior margin of skull roof

1 close to posterior margin of skull roof

176. Number of extrascapulars

0 uneven

1 paired

177. Dermal neck-joint between paired main-lateral-line-bearing bones of skull and shoulder girdle

0 absent

1 present

178. Foramina (similar to infradentary foramina) on cheek bones

0 absent

1 present

179. Lacrimal posteriorly enclosing posterior nostril

0 absent

1 present

180. Most posterior major bone of cheek bearing preopercular canal (preopercular) extending forward, close to orbit

0 absent

1 present

181. Number of cheek bones bearing preopercular canal posterior to jugal

0 one

1 two

182. Bone bearing both quadratojugal pit-line and preopercular canal

0 absent

1 present

183. Dermohyal

0 absent

1 present

184. Premaxillae with inturned symphysial processes

0 absent

1 present

185. Premaxilla forming part of orbit

0 absent

1 present

186. Preorbital process of premaxilla

0 absent

1 present

187. Ventral margin of maxilla

0 straight

1 curved

188. Course of ethmoid commissure

0 middle portion through median rostral

1 sutural course

2 through bone center of premaxillary

189. Position of anterior pit-line

0 on paired median skull roofing bones over the otico-occipital division of braincase

1 on paired median skull roofing bones over the sphenoid division of braincase

190. Middle and posterior pit-lines on postparietal

0 posteriorly situated

1 mesially situated

191. Position of middle and posterior pit lines

0 close to midline

1 near the central portion of each postparietal

192. Course of supraorbital canal

0 between anterior and posterior nostrils

1 anterior to both nostrils

193. Course of supraorbital canal

0 straight

1 lyre-shaped

194. Posterior end of supraorbital canal

0 in postparietal

1 in parietal

2 in intertemporal

3 in nuchal plate

4 in postpineal plate

195. Contact between otic and supraorbital canals

0 not in contact

1 in contact

196. Contact of supraorbital and infraorbital canals

0 in contact rostrally

1 not in contact rostrally

197. Otic canal

0 runs through skull roof

1 follows edge of skull roof

198. Infraorbital canal follows premaxillary suture

0 no

1 yes

199. Sensory canal or pit-line associated with maxilla

0 absent

1 present

200. Anterior portion of preopercular canal

0 present

1 absent

201. Foramen in hyomandibular

0 absent

1 present

202. Large dermal plates forming outer dental arcade

0 only with denticles

1 with large monolinear tooth row

203. Tooth-bearing median rostral

0 absent

1 present

204. Teeth of dentary

0 reaching anterior end of dentary

1 not reaching anterior end

205. Marginal denticle band on coronoids

0 broad band, at least posteriorly

1 narrow band with 2-4 denticle rows

206. Infradentary

0 absent

1 present

207. Infradentary foramina

0 present

1 absent

208. Large ventromesially directed flange of symphysial region of mandible

0 absent

1 present

209. Flange like extension of mandible composed of prearticular and Meckelian ossification

0 absent

1 present

210. Strong ascending flexion of symphysial region of mandible

0 absent

1 present

211. Parasymphysial plate

0 detachable tooth whorl

1 long with posterior corner, sutured to coronoid, denticulated or with tooth row

2 absent

212. Anterior end of prearticular

0 far from jaw symphysis

1 near jaw symphysis

213. Prearticular - dentary contact

0 present

1 absent

214. Meckelian bone exposed immediately anterior to first coronoid

0 yes

1 no

215. Dermal plates on mesial (lingual) surfaces of Meckels cartilage and palatoquadrate

0 absent

1 present

216. Biconcave glenoid on lower jaw

0 absent

1 present

217. Course of mandibular canal

0 not passing through most posterior infradentary

1 passing through most posterior infradentary

218. Course of mandibular canal

0 passing through dentary

1 not passing through dentary

219. Fenestra ventrolateralis

0 absent

1 present

2 common ventral fenestra for anterior and posterior nostrils

220. Developed postorbital cavity

0 absent

1 present

221. Unconstricted cranial notochord

0 absent

1 present

222. Descending process of sphenoid (with its posterior extremity lacking periostegeal lining)

0 absent

1 present

223. Hyoid arch articulation

0 on lateral commissure

1 on otic capsule wall

224. Opercular suspension on braincase

0 absent

1 present

225. Vomerine fangs

0 absent

1 present

226. Vomeral area with grooves and raised areas

0 absent

1 present

227. Parasphenoid

0 protruding forward into ethmoid region of endocranium

1 behind ethmoid region

228. Denticulated field of parasphenoid

0 without spiracular groove

1 with spiracular groove

229. Parasphenoid denticle field with anteriorly divergent lateral margins

0 absent

1 present

230. Parasphenoid denticle field

0 terminates at or anterior to level of foramina for internal carotid arteries

1 extends posterior to foramina for internal carotid arteries

231. Presupracleithrum

0 absent

1 present

232. Anocleithrum

0 element developed as postcleithrum

1 element developed as anocleithrum sensu stricto

233. Endoskeletal supports in pectoral fin

0 multiple elements articulating with girdle

1 single element ("humerus") articulating with girdle

234. Dorsal cleithrum (AL of the Placodermi), ventral cleithrum (AVL of the Placodermi) and pectoral spine (SP of the Placodermi)

0 not fused

1 fused

235. Relationship of clavicle to cleithrum

0 ascending process of clavicle overlapping cleithrum laterally

1 ascending process of clavicle wrapping round anterior edge of cleithrum, overlapping it both laterally and mesially

236. Triradiate scapulocoracoid

0 absent

1 present

237. Subscapular foramen

0 absent

1 present

238. Pectoral propterygium

0 absent

1 present

239. Pelvic girdle with substantial dermal component

0 yes

1 no

240. Pelvic fin spine

0 absent

1 present

241. Number of sclerotic plates

0 four or less

1 more than four

242. Ethmoid articulation for palatoquadrate

0 placed on postnasal wall

1 extends posteriorly to the level of N.II

243. Contact between palatoquadrate and dermal cheek bones

0 continuous contact of metapterygoid and autopalatine

1 metapterygoid and autopalatine contacts separated by gap between commissural lamina of palatoquadrate and cheek bones

244. Metapterygoid with developed medial ventral protrusion

0 absent

1 present

245. Internasal pits

0 absent

1 undifferentiated or anterior palatal fossa

2 shallow, paired pits with strong midline ridge

3 deep, peer-shaped pits

246. jaws

0 absent

1 present

247. Optic fissure

0 present

1 absent

248. Central dermal skull bone (nuchal) with converging posterior pit-line canals and supraorbital canals

0 absent

1 converging but not meeting

2 crossing as an X in bone

249. Deep, high supragnathal bone with durophagous occlusal surface

0 absent

1 present

250. Intromittent organ with one large J-shaped element

0 absent

1 present

251. Intromittent organ ('clasper') consisting entirely of cartilage, formed from distal part of pelvic fin

0 absent

1 present

252. Fringing fulcra

0 absent

1 present

253. Prismatic calcified cartilage

0 single layered

1 multi-layered

254. Enamel(oid) present on dermal bones and scales

0 absent

1 present

255. Enamel

0 single-layered

1 multi-layered

256. Enamel layers

0 applied directly to one another (ganoine)

1 separated by layers of dentine

257. Extensive pore canal network

0 absent

1 present

258. Vertical canal associated with preopercular/suborbital canal

0 absent

1 present

259. Bone cell lacunae in body scale bases

0 present

1 absent

260. Main dentinous tissue forming fin spine

0 osteodentine

1 orthodentine

261. Differentiated lepidotrichia

0 absent

1 present

262. Profile of scales with constriction between crown and base

0 neck similar in width to crown

1 neck greatly constricted, resulting in anvil-like shape

263. Basal pore in scales

0 absent

1 present

264. Scute-like ridge scales (basal fulcra)

0 absent

1 present

265. Dermal ornamentation

0 smooth

1 parallel, vermiform ridges

2 concentric ridges

3 tuberculate

266. Sensory canals/grooves

0 contained within the thickness of dermal bones

1 contained in prominent ridges on visceral surface of bone

267. Anterior pit line of dermal skull roof

0 absent

1 present

268. Cranial spines

0 absent

1 present, multicuspid

2 present, monocuspid

269. Endolymphatic duct relationship to median skull roof bone (i.e. nuchal plate)

0 within median bone

1 on bones flanking the median bone (e.g. paranuchals)

270. Dermal plate associated with pineal eminence or foramen

0 contributes to orbital margin

1 plate bordered laterally by skull roofing bones

271. Broad supraorbital vaults

0 absent

1 present

272. Median commisure between supraorbital sensory lines

0 absent

1 present

273. Otic canal extends through postparietals

0 absent

1 present

274. Suture between paired skull roofing bones (centrals of placoderms; postparietals of osteichthyans)

0 straight

1 sinusoidal

275. Medial processes of paranuchal wrapping posterolateral corners of nuchal plate

0 absent

1 present

2 paranuchals precluded from nuchal by centrals

3 no median posterior skull roof bone

276. Paired pits on ventral surface of nuchal plate

0 absent

1 present

277. Sclerotic ring

0 absent

1 present

278. Cheek plate

0 undivided

1 divided (i.e., squamosal and preopercular)

279. Subsquamosals in taxa with divided cheek

0 absent

1 present

280. Preopercular shape

0 rhombic

1 bar-shaped

281. Gill arches

0 largely restricted to region under braincase

1 extend far posterior to braincase

282. Hypohyal

0 absent

1 present

283. Endoskeletal urohyal

0 absent

1 present

284. Enamel(oid) on teeth

0 absent

1 present

285. Distribution of tooth whorls

0 upper and lower jaws

1 lower jaws only

2 upper jaws only

286. Premaxilla

0 extends under orbit

1 restricted anterior to orbit

287. Pair of tooth plates (anterior supragnathals or vomers) on ethmoidal plate

0 absent

1 present

288. Extent of infradentaries

0 along much of ventral margin of dentary

1 restricted to posterior half of dentary

289. Position of upper mandibular arch cartilage (and associated cheek plate where present)

0 entirely suborbital

1 with a postorbital extension

290. Autopalatine and quadrate

0 comineralized

1 separate mineralizations

291. Palatoquadrate fused with neurocranium

0 absent

1 present

292. Buccohypophysial canal in parasphenoid

0 single

1 paired

293. Transverse otic process

0 present

1 absent

294. Jugular canal

0 long (invested in otic region along length of skeletal labyrinth)

1 short (restricted to region anterior of skeletal labyrinth)

2 absent (jugular vein uninvested in otic region)

295. Postorbital process

0 absent

1 present

296. Canal for jugular in postorbital process

0 absent

1 present

297. Series of perforations for innervation of supraorbital sensory canal in supraorbital shelf

0 absent

1 present

298. Subcranial ridges

0 absent

1 present

299. Horizontal semicircular canal in dorsal view

0 medial to path of jugular vein

1 dorsal to jugular vein

300. Synotic tectum

0 absent

1 present

301. Shape of median dorsal ridge anterior to endolymphatic fossa

0 developed as a squared-off ridge or otherwise ungrooved

1 bears a midline groove

302. Branchial ridges

0 present

1 reduced to vagal process

2 absent (articulation made with bare cranial wall)

303. Craniospinal process ("supravagal process" in Stensio)

0 absent

1 present

304. Stalk-shaped parachordal/occipital region

0 absent

1 present

305. Paired occipital facets

0 absent

1 present

306. Size of aperture to notochordal canal

0 much smaller than foramen magnum

1 as large, or larger, than foramen magnum

307. Shape of dorsal blade of dermal shoulder girdle

0 spatulate

1 pointed

308. Posterior dorsolateral plate or equivalent

0 absent

1 present

309. Scapular infundibulum

0 absent

1 present

310. Number of basals in polybasal pectoral fins

0 three or more

1 two

311. Number of mesomeres in metapterygial axis

0 five or fewer

1 seven or more

312. Biserial pectoral fin endoskeleton

0 absent

1 present

313. Filamentous extension of pectoral fin from axillary region

0 absent

1 present

314. Supraneurals in axial lobe of caudal fin

0 absent

1 present

315. Fin spine cross-section

0 round or horseshoe shaped

1 Flat-sided, with rectangular profile

316. Intermediate spines when present

0 one pair

1 multiple pairs

317. Expanded spine rib on leading edge of spine

0 absent

1 present

318. Spine ridges

0 converging at the distal apex of the spine

1 converging on leading edge of spine

319. Series of thoracic supraneurals

0 absent

1 present

320. Posterior dorsal fin shape

0 base approximately as broad as tall, not broader than all of other median fins

1 base much longer than the height of the fin, substantially longer than any of the other dorsal fins

321. Basal plate in dorsal fin

0 absent

1 present

322. Branching radial structure articulating with dorsal fin basal plate

0 absent

1 present

323. Basal plate in anal fin

0 absent

1 present

324. Relative position of jugular groove and hyomandibular articulation:

0 hyomandibula dorsal or same level (i.e. on bridge)

1 jugular vein passing dorsal or lateral to hyomandibula

325. Canal-bearing bone of skull roof extends far past posterior margin of parietals. 0 no

1 yes

326. Position of anterior pitline

0 on postparietal

1 on parietal

327. Opening in dermal skull roof for spiracular bounded by bones carrying otic canal

0 absent

1 present

328. Preoperculosubmandibular

0 absent

1 present

329. Urohyal shape

0 absent

1 Vertical plate

330. Extensive flange composed of prearticular and Meckelian bone that extends beyond ventral edge of outer dermal series

0 absent

1 present

331. Inturned medial process of premaxilla

0 absent

1 present

332. Posterior nostril

0 facial

1 at margin oral cavity

2 palatal

333. Size of profundus canal in postnasal wall

0 samall

1 large

334. Paired pineal and parapineal tracts

0 absent

1 present

335. Endoskeletal spiracular canal

0 open

1 partial enclosure or spiracular bar

2 complete enclosure in canal

336. Relationship of crown and base of isolated trunk scale

0 crown fully covering the base

1 crown sitting on the bony base, with an exposed depressed field overlapped by adjacent scale in articulation

337. Optic lobes

0 narrower than cerebellum

1 same width or wider than cerebellum

338 Crus commune of anterior and posterior semicircular can

0 dorsal to endocranial roof

1 ventral to endocranial roof

339. Cranial cavity and labyrinth

0 widely spaced

1 closely spaced

340. Horizontal semicircular canal

0 absent

1 present

341 Supraotic cavity

0 absent

1 present

342 Pelvic fin

0 monobasal

1 polybasal

343 Keel of scale

0 absent

1 present

344 Posterior ledge (or secondary keel) of scale

0 absent

1 weak

2 developed

345 Anteroventral process of scale

0 absent

1 present

346 Ventral process of scale

0 present

1 absent

Supplementary References

Ahlberg PE. 1989. Paired fin skeletons and relationships of the fossil group Porolepiformes (Osteichthyes: Sarcopterygii). Zoological Journal of the Linnean Society 96:119-166.

Anderson ME, Hiller N, Gess RW. 1994. The first *Bothriolepis*-associated Devonian fish fauna from

Africa. South African Journal of Science 90:397-403.

Andrews SM, Long JA, Ahlberg PE, Barwick R, Campbell KSW. 2005. The structure of the sarcopterygian *Onychodus jandemarrai* n. sp. from Gogo, Western Australia: with a functional interpretation of the skeleton. Transactions of the Royal Society of Edinburgh: Earth Sciences 96:197- 307.

Andrews SM, Westoll TS. 1970. The postcranial skeleton of rhipidistian fishes excluding *Eusthenopteron*. Transactions of the Royal Society of Edinburgh: Earth Sciences 68:391-489.

Arratia G, Cloutier R. 1996. Reassessment of the morphology of *Cheirolepis canadensis* (Actinopterygii). In: Schultze H-P, Cloutier R, editors. Devonian Fishes and Plants of Miguasha, Quebec, Canada. München: Verlag Dr. Friedrich Pfeil. p 165-197.

Arratia G, Cloutier R. 2004. A new cheirolepidid fish from the Middle-Upper Devonian of Red Hill, Nevada, USA. In: Arratia G, Wilson MVH, Cloutier R, editors. Recent Advances in the Origin and Early Radiation of Vertebrates. München: Verlag Dr. Friedrich Pfeil. p 583-598.

Arsenault M, Desbiens S, Janvier P, Kerr J. 2004. New data on the soft tissues and external morphology of the antiarch *Bothriolepis canadensis* (Whiteaves, 1880) from the Upper Devonian of Miguasha, Quebec. In: Arratia G, Wilson MVH, Cloutier R, editors. Recent Advances in the Origin and Early Radiation of Vertebrates. München: Verlag Dr. Friedrich Pfeil. p 439-454.

Basden AM, Young GC. 2001. A primitive actinopterygian neurocranium from the Early Devonian of southeastern Australia. Journal of Vertebrate Paleontology 21:754-766.

Basden AM, Young GC, Coates MI, Ritchie A. 2000. The most primitive osteichthyan braincase? Nature 403:185-188.

Bendix-Almgreen SE. 1975. The paired fins and shoulder girdle in *Cladoselache*, their morphology and phyletic significance. In: Lehman JP, editor. Problèmes actuels de Paléontologie-Evolution des Vertébrés. Paris: Colloques Internationaux du Centre National de la Recherche Scientifique. p 111-123.

Bernacsek GM, Dineley DL. 1977. New acanthodians from the Delorme Formation (Lower Devonian) of N.W.T., Canada. Palaeontographica Abteilung A 158:1-25.

Botella H, Blom H, Dorka M, Ahlberg PE, Janvier P. 2007. Jaws and teeth of the earliest bony fishes. Nature 448:583-586.

Brazeau MD. 2009. The braincase and jaws of a Devonian 'acanthodian' and modern gnathostome origins. Nature 457:305-308.

Brazeau MD, de Winter V. 2015. The hyoid arch and braincase anatomy of *Acanthodes* support

chondrichthyan affinity of 'acanthodians'. Proceedings of the Royal Society B: Biological Sciences 282:20152210.

Burrow CJ. 1994. Form and function in scales of *Ligulalepis toombsi* Schultze, a palaeoniscoid from the Early Devonian of Australia. Records of the Southern Australian Museum 27:175-185.

Burrow CJ. 1995. A new lophosteiform (Osteichthyes) from the Lower Devonian of Australia. Geobios 19:327-333.

Burrow CJ, Turner S. 1998. Devonian placoderm scales from Australia. Journal of Vertebrate Paleontology 18:677-695.

Burrow CJ, Turner S. 1999. A review of placoderm scales, and their significance in placoderm phylogeny. Journal of Vertebrate Paleontology 19:204-219.

Burrow CJ, Young GC. 2012. New Information on *Culmacanthus* (Acanthodii: Diplacanthiformes) from the ?Early–Middle Devonian of southeastern Australia. Proceedings of the Linnean Society of New South Wales 134:21-29.

Carr RK, Johanson Z, Ritchie A. 2009. The phyllolepid placoderm *Cowralepis mclachlani*: Insights into the evolution of feeding mechanisms in jawed vertebrates. Journal of Morphology 270:775-804.

Chang M-M. 1982. The braincase of *Youngolepis*, a Lower Devonian crossopterygian from Yunnan, south-western China. Stockholm: University of Stockholm, Department of Geology.

Chang M-M. 1991. Head exoskeleton and shoulder girdle of *Youngolepis*. In: Chang M-M, Liu Y-H, Zhang G-R, editors. Early Vertebrates and Related Problems of Evolutionary Biology. Beijing: Science Press. p 355-378.

Chang M-M. 1995. *Diabolepis* and its bearing on the relationships between porolepiforms and dipnoans. Bulletin du Muséum national d'Histoire naturelle, Paris 4e sér, Section C 17:235-268.

Chang M-M. 2004. Synapomorphies and scenarios - more characters of *Youngolepis* betraying its affinity to the Dipnoi. In: Arratia G, Wilson MVH, Cloutier R, editors. Recent Advances in the Origin and Early Radiation of Vertebrates. München: Verlag Dr. Friedrich Pfeil. p 665-686.

Chang M-M, Smith MM. 1992. Is *Youngolepis* a porolepiform? Journal of Vertebrate Paleontology 12:294-312.

Chang M-M, Yu X-B. 1981. A new crossopterygian, *Youngolepis praecursor*, gen. et sp. nov., from Lower Devonian of E. Yunnan, China. Scientia Sinica 24:89-99.

Chang M-M, Yu X-B. 1984. Structure and phylogenetic significance of *Diabolichthys speratus* gen.

et sp. nov., a new dipnoan-like form from the Lower Devonian of eastern Yunnan, China. Proceedings of the Linnean Society of New South Wales 107:171-184.

Chang M-M, Zhu M. 1993. A new Middle Devonian osteolepidid from Qujing, Yunnan. Memoirs of the Association of Australasian Palaeontologists 15:183-198.

Choo B. 2012. Revision of the actinopterygian genus *Mimipiscis* (=*Mimia*) from the Upper Devonian Gogo Formation of Western Australia and the interrelationships of the early Actinopterygii. Transactions of the Royal Society of Edinburgh: Earth and Environmental Science 102:77-104.

Choo B, Zhu M, Qu Q-M, Yu X-B, Jia L-T, Zhao W-J. 2017. A new osteichthyan from the late Silurian of Yunnan, China. PloS One 12:e0170929.

Clément G. 2004. Nouvelles données anatomiques et morphologie générale des 'Porolepidae' (Dipnomorpha, Sarcopterygii). Revue de Paléobiologie, Genère 9:193-211.

Clément G, Ahlberg PE. 2010. The endocranial anatomy of the early sarcopterygian *Powichthys* from Spitsbergen, based on CT scanning. In: Elliott DK, Maisey JG, Yu X-B, Miao D-S, editors. Morphology, Phylogeny and Paleobiogeography of Fossil Fishes. München: Verlag Dr. Friedrich Pfeil. p 363-377.

Clément G, Janvier P. 2004. *Powichthys spitsbergensis* sp. nov., a new member of the Dipnomorpha (Sarcopterygii, lobe-finned fishes) from the Lower Devonian of Spitsbergen, with remarks on basal dipnomorph anatomy. Fossils & Strata 50:92-112.

Cloutier R. 1996. The primitive actinistian *Miguashaia bureaui* Schultze (Sarcopterygii). In: Schultze H-P, Cloutier R, editors. Devonian Fishes and Plants of Miguasha, Quebec, Canada. München: Verlag Dr. Freidrich Pfeil. p 227-247.

Coates MI, Davis S. 2010. About the ears: *Acanthodes* re-examined and gnathostome origin re-analyzed. Journal of Vertebrate Paleontology 30 (supplement):74A.

Coates MI, Gess RW. 2007. A new reconstruction of *Onychoselache* Traquairi, comments on early chondrichthyan pectoral girdles and hybodontiform phylogeny. Palaeontology 50:1421-1446.

Coates MI, Sequeira SEK. 1998. The braincase of a primitive shark. Transactions of the Royal Society of Edinburgh: Earth Sciences 89:63-85.

Coates MI, Sequeira SEK. 2001a. Early sharks and primitive gnathostome interrelationships. In: Ahlberg PE, editor. Major Events in Early Vertebrate Evolution: Palaeontology, Phylogeny, Genetics and Development. London: Taylor & Francis. p 241-262.

Coates MI, Sequeira SEK. 2001b. A new stethacanthid chondrichthyan from the Lower Carboniferous of Bearsden, Scotland. Journal of Vertebrate Paleontology 21:438-459.

Coates MI, Sequeira SEK, Sansom IJ, Smith MM. 1998. Spines and tissues of ancient sharks. Nature 396:729-730.

Davis SP, Finarelli JA, Coates MI. 2012. *Acanthodes* and shark-like conditions in the last common ancestor of modern gnathostomes. Nature 486:247-250.

Denison RH. 1978. Placodermi. In: Schultze H-P, editor. Handbook of Paleoichthyology, vol. 2. Stuttgart: Gustav Fischer Verlag. p 128.

Denison RH. 1979. Acanthodii. Stuttgart: Gustav Fischer Verlag.

Dennis-Bryan K. 1987. A new species of eastmanosteid arthrodire (Pisces: Placodermi) from Gogo, Western Australia. Zoological Journal of the Linnean Society 90:1-64.

Dennis K, Miles RS. 1981. A pachyosteomorph arthrodire from Gogo, Western Australia. Zoological Journal of the Linnean Society 73:213 ~ 258.

Dick JRF, Maisey JG. 1980. The Scottish Lower Carboniferous shark *Onychoselache traquairi*. Palaeontology 23:363-374.

Downs JP, Donoghue PCJ. 2009. Skeletal histology of *Bothriolepis canadensis* (Placodermi, Antiarchi) and evolution of the skeleton at the origin of jawed vertebrates. Journal of Morphology 270:1364-1380.

Dupret V. 2010. Revision of the genus *Kujdanowiaspis* Stensiö, 1942 (Placodermi, Arthrodira, "Actinolepida") from the Lower Devonian of Podolia (Ukraine). Geodiversitas 32:5-63.

Dupret V, Sanchez S, Goujet D, Tafforeau P, Ahlberg PE. 2014. A primitive placoderm sheds light on the origin of the jawed vertebrate face. Nature 507:500-503.

Dupret V, Zhu M. 2008. The earliest phyllolepid (Placodermi, Arthrodira) from the Late Lochkovian (Early Devonian) of Yunnan (South China). Geological Magazine 145:257-278.

Forey PL. 1998. History of the Coelacanth Fishes. London: Chapman & Hall.

Forey PL, Ahlberg PE, Luksevics E, Zupins I. 2000. A new coelacanth from the Middle Devonian of Latvia. Journal of Vertebrate Paleontology 20:243-252.

Gagnier P-Y. 1996. Acanthodii. In: Schultze H-P, Cloutier R, editors. Devonian Fishes and Plants of Miguasha, Quebec, Canada. München: Verlag Dr. Friedrich Pfeil. p 149-164.

Gagnier PY. 1995. Ordovician vertebrates and agnathan phylogeny. Bulletin du Muséum national d'Histoire naturelle, Paris 4e sér, Section C 17:1-37.

Gagnier PY, Hanke GF, Wilson MVH. 1999. *Tetanopsyrus lindoei* gen. et sp. nov., an Early Devonian acanthodian from the Northwest Territories, Canada. Acta Geologica Polonica 49:81-96.

Gagnier PY, Wilson MVH. 1996a. Early Devonian acanthodians from northern Canada.

Palaeontology 39:241-258.

Gagnier PY, Wilson MVH. 1996b. An unusual acanthodian from northern Canada: revision of *Brochoadmones milesi*. Modern Geology 20:235-251.

Gardiner BG. 1984. The relationships of the palaeoniscid fishes, a review based on new specimens of *Mimia* and *Moythomasia* from the Upper Devonian of Western Australia. Bulletin of the British Museum (Natural History), Geology 37:173-428.

Gardiner BG, Miles RS. 1994. Eubrachythoracid arthrodires from Gogo, Western Australia. Zoological Journal of the Linnean Society 112:443-477.

Giles S, Friedman M, Brazeau MD. 2015. Osteichthyan-like cranial conditions in an Early Devonian stem gnathostome. Nature 520:82-85.

Giles S, Rücklin M, Donoghue PCJ. 2013. Histology of "placoderm" dermal skeletons: Implications for the nature of the ancestral gnathostome. Journal of Morphology 274:627-644.

Goujet DF. 1973. *Sigaspis*, un nouvel arthrodire du Dévonien inférieur du Spitsberg. Palaeontographica Abteilung A 143:73-88.

Goujet DF. 1975. *Dicksonosteus*, un nouvel arthrodire du Dévonien du Spitsberg remarques sur le squelette visceral des Dolichothoraci. In: Lehman JP, editor. Problèmes actuels de Paléontologie-Evolution des Vertébrés. Paris: Colloques Internationaux du Centre National de la Recherche Scientifique. p 81-99.

Goujet DF. 1984. Les poissons placodermes du Spitsberg. Arthrodires Dolichothoraci de la Formation de Wood Bay (Dévonien inférieur). Paris: Editions Centre National Recherche Scientifique, Cahiers de Paléontologie.

Goujet DF, Young GC. 2004. Placoderm anatomy and phylogeny: new insights. In: Arratia G, Wilson MVH, Cloutier R, editors. Recent Advances in the Origin and Early Radiation of Vertebrates. München: Verlag Dr. Friedrich Pfeil. p 109-126.

Grogan ED, Lund R. 2000. *Debeerius ellefseni* (fam. nov., gen. nov., spec. nov.), an autodiastylic chondrichthyan from the Mississippian bear gulch limestone of Montana (USA), the relationships of the chondrichthyes, and comments on gnathostome evolution. Journal of Morphology 243:219-245.

Gross W. 1935. Histologische Studien am Aussenskelett fossiler Agnathen und Fische. Palaeontographica Abteilung A 83:1-60.

Gross W. 1937. Das Kopfskelett von *Cladodus wildungensis*, 1. Endocranium und Palatoquadratum. Senckenbergiana 19:80-107.

Gross W. 1938. Das Kopfskelett von *Cladodus wildungensis* Jaekel. 2, Teil: Der Kieferbogen.

Anhang: *Protacrodus vetustus* Jaekel. Senckenbergiana 20:123-145.

Gross W. 1961. *Lunaspis broilii* und *Lunaspis heroldi* aus dem Hunsrückschiefer (Unterdevon, Rheinland). Notizblatt des hessisches Landesanstalt für Bodenforschung 89:17-43.

Gross W. 1963. *Gemuendina stuertzi* Traquair. Neuuntersuchung. Notizblatt des hessisches Landesanstalt für Bodenforschung 91:36-73.

Gross W. 1969. *Lophosteus superbus* Pander, ein Teleostome aus dem Silur Oesels. Lethaia 2:15-47.

Gross W. 1971. *Lophosteus superbus* Pander: Zähne, zahnknochen und besondere schuppenformen. Lethaia 4:131-152.

Halstead LB. 1979. Internal anatomy of the polybranchiaspids (Agnatha, Galeaspida). Nature 282:833-836.

Hanke GF. 2008. *Promesacanthus eppleri* n. gen., n. sp., a mesacanthid (Acanthodii, Acanthodiformes) from the Lower Devonian of northern Canada. Geodiversitas 30:287-302.

Hanke GF, Davis SP. 2008. Redescription of the acanthodian *Gladiobranchus probaton* Bernacsek & Dineley, 1977, and comments on diplacanthid relationships. Geodiversitas 30:303-330.

Hanke GF, Davis SP. 2012. A re-examination of *Lupopsyrus pygmaeus* Bernacsek & Dineley, 1977 (Pisces, Acanthodii). Geodiversitas 34:469-487.

Hanke GF, Davis SP, Wilson MVH. 2001. New species of the acanthodian genus *Tetanopsyrus* from northern Canada, and comments on related taxa. Journal of Vertebrate Paleontology 21:740-753.

Hanke GF, Wilson MVH. 2004. New teleostome fishes and acanthodian systematics. In: Arratia G, Wilson MVH, Cloutier R, editors. Recent Advances in the Origin and Early Radiation of Vertebrates. München: Verlag Dr. Friedrich Pfeil. p 189-216.

Hanke GF, Wilson MVH. 2006. Anatomy of the Early Devonian acanthodian *Brochoadmones milesi* based on nearly complete body fossils, with comments on the evolution and development of paired fins. Journal of Vertebrate Paleontology 26:526-537.

Hanke GF, Wilson MVH. 2010. The putative stem-group chondrichthyans *Kathemacanthus* and *Seretolepis* from the Lower Devonian MOTH locality, Mackenzie Mountains, Canada. In: Elliott DK, Maisey JG, Yu X-B, Miao D-S, editors. Morphology, Phylogeny and Paleobiogeography of Fossil Fishes. Miinchen: Verlag Dr. Friedrich Pfeil. p 159-182.

Heidtke U. 1982. Der Xenacanthidae *Orthacanthus senckenbergianus* aus dem pfälzischen Rotliegended (Unter-Perm). Polichia 70:65-86.

Heidtke U. 1998. Revision der Gattung *Orthacanthus* Agassiz 1843 (Chondrichthyes: Xenacanthida). Paläontologische Zeitschrift 72:135-147.

Heintz A. 1937. Die Downtonischen und Devonischen Vertebraten von Spitzbergen VI. *Lunaspis*-arten aus dem Devon Spitzbergens. Skrifter om Svalbard og Ishavet 72:1-23.

Hemmings SK. 1978. The Old Red Sandstone antiarchs of Scotland: *Pterichthyodes* and *Microbrachius*. Monographs of the Palaeontographical Society 131:1-64.

Hermus CR. 2003. Taxonomy and ontogeny of *Ischnacanthus* (Pisces: Acanthodii: Ischnacanthiformes) from the Lower Devonian (Lochkovian), Northwest Territories, Canada. In: Department of Biological Sciences. Edmonton: University of Alberta. p 255.

Holland T, Long JA. 2009. On the phylogenetic position of *Gogonasus andrewsae* Long 1985, within the Tetrapodomorpha. Acta Zoologica 90:285-296.

Janvier P. 1981. The phylogeny of the Craniata, with particular reference to the significance of fossil "agnathans". Journal of Vertebrate Paleontology 1:121-159.

Janvier P. 1985. Les Céphalaspides du Spitsberg: anatomie, phylogénie et systématique des Ostéostracés siluro-dévoniens; revisions des Ostéostracés de la Formation de Wood Bay (Dévonien inférieur du Spitsberg). Paris: Cahiers de Paléontologie, Centre national de la Recherche scientifique.

Janvier P. 1996. Early Vertebrates. Oxford: Clarendon Press.

Janvier P, Arsenault M, Desbiens S. 2004. Calcified cartilage in the paired fins of the osteostracan *Escuminaspis laticeps* (Traquair 1880), from the Late Devonian of Miguasha (Québec, Canada), with a consideration of the early evolution of the pectoral fin endoskeleton in vertebrates. Journal of Vertebrate Paleontology 24:773-779.

Jarvik E. 1948. Note on the Upper Devonian vertebrate fauna of East Greenland and on the age of the ichthyostegid stegocephalians. Arkiv för Zoologi 41:1-8.

Jarvik E. 1972. Middle and Upper Devonian Porolepiformes from East Greenland with special reference to *Glyptolepis groenlandica* n. sp., and a discussion on the structure of the head in the Porolepiformes. Meddelelser om Grønland 187:1-307.

Jarvik E. 1977. The systematic position of acanthodian fishes. In: Andrews SM, Miles RS, Walker AD, editors. Problems in Vertebrate Evolution. London: Academic Press. p 199-225.

Jarvik E. 1980. Basic Structure and Evolution of Vertebrates, Vol. 1. London: Academic Press.

Jessen HL. 1966. Die Crossopterygier des Oberen Plattenkalkes (Devon) der Bergisch-Gladbach-Paffrather Mulde (Rheinisches Schiefergebirge) unter Berücksichtigung von amerikanischem und europäischem *Onychodus*-material. Arkiv för Zoologi 18:305-389.

Jessen HL. 1975. A new choanate fish, *Powichthys thorsteinssoni* n.g., n.sp., from the early Lower Devonian of the Canadian Arctic Archipelago. In: Lehman JP, editor. Problèmes actuels de

Paléontologie-Evolution des Vertébrés. Paris: Colloques Internationaux du Centre National de la Recherche Scientifique. p 213-222.

Jessen HL. 1980. Lower Devonian Porolepiformes from the Canadian Arctic with special reference to *Powichthys thorsteinssoni* Jessen. Palaeontographica Abteilung A 167:180-214.

Johanson Z. 1997. New *Remigolepis* (Placodermi; Antiarchi) from Canowindra, New South Wales, Australia. Geological Magazine 134:813-846.

Johanson Z, Smith MM. 2005. Origin and evolution of gnathostome dentitions: a question of teeth and pharyngeal denticles in placoderms. Biological Reviews 80:303-345.

Liu T-S, P'an K. 1958. Devonian fishes from Wutung Series near Nanking, China. Palaeontologica Sinica, new series C 141:1-41.

Liu Y-H. 1973. On the new forms of Polybranchiaspiformes and Petalichthyida from Devonian of Southwest China. Vertebrata PalAsiatica 11:132-143.

Liu Y-H. 1991. On a new petalichthyid, *Eurycaraspis incilis* gen. et sp. nov., from the Middle Devonian of Zhanyi, Yunnan. In: Chang M-M, Liu Y-H, Zhang G-R, editors. Early Vertebrates and Related Problems of Evolutionary Biology. Beijing: Science Press. p 139-177.

Long JA. 1983. A new diplacanthoid acanthodian from the Late Devonian of Victoria. Memoir of the Association of Australasian Palaeontologists 1:51-65.

Long JA. 1985. A new osteolepidid fish from the Upper Devonian Gogo Formation, western Australia. Records of the Western Australian Museum 12:361-377.

Long JA. 1988. New palaeoniscoid fishes from the Late Devonian and Early Carboniferous of Victoria. Memoirs of the Association of Australasian Palaeontologists 7:1-64.

Long JA. 1997. Ptyctodontid fishes (Vertebrata, Placodermi) from the Late Devonian Gogo Formation, Western Australia, with a revision of the European genus *Ctenurella* Ørvig, 1960. Geodiversitas 19:515-555.

Long JA. 2001. On the relationships of *Psarolepis* and the onychodontiform fishes. Journal of Vertebrate Paleontology 21:815-820.

Long JA, Barwick RE, Campbell KSW. 1997. Osteology and functional morphology of the osteolepiform fish *Gogonasus andrewsae* Long, 1985, from the Upper Devonian Gogo Formation, Western Australia. Records of the Western Australian Museum, Supplement 53:1-89.

Long JA, Mark-Kurik E, Johanson Z, Lee MS, Young GC, Zhu M, Ahlberg PE, Newman M, Jones R, Blaauwen JD, Choo B, Trinajstic K. 2015. Copulation in antiarch placoderms and the

origin of gnathostome internal fertilization. Nature 517:196-199.

Long JA, Mark-Kurik E, Young GC. 2014. Taxonomic revision of buchanosteoid placoderms (Arthrodira) from the Early Devonian of south-eastern Australia and Arctic Russia. Australian Journal of Zoology 62:26.

Long JA, Trinajstic K, Johanson Z. 2009. Devonian arthrodire embryos and the origin of internal fertilization in vertebrates. Nature 457:1124-1127.

Long JA, Trinajstic K, Young GC, Senden T. 2008. Live birth in the Devonian period. Nature 453:650-652.

Long JA, Young GC, Holland T, Senden TJ, Fitzgerald EMG. 2006. An exceptional Devonian fish from Australia sheds light on tetrapod origins. Nature 444:199-202.

Lu J, Giles S, Friedman M, den Blaauwen JL, Zhu M. 2016. The oldest actinopterygian highlights the cryptic early history of the hyperdiverse ray-finned fishes. Current Biology 26:1602-1608.

Lu J, Giles S, Friedman M, Zhu M. 2017. A new stem sarcopterygian illuminates patterns of character evolution in early bony fishes. Nature Communications 8:1932.

Maisey JG. 1980. An evaluation of jaw suspension in sharks. American Museum Novitates 2706:1-17.

Maisey JG. 1989a. *Hamiltonichthys mapesi*, g. & sp. nov. (Chondrichthyes; Elasmobranchii), from the Upper Pennsylvanian of Kansas. American Museum Novitates 2931:1-42.

Maisey JG. 1989b. Visceral skeleton and musculature of a late Devonian shark. Journal of Vertebrate Paleontology 9:174-190.

Maisey JG. 2001. A primitive chondrichthyan braincase from the Middle Devonian of Bolivia. In: Ahlberg PE, editor. Major Events in Early Vertebrate Evolution: Palaeontology, Phylogeny, Genetics and Development. London: Taylor & Francis. p 263-288.

Maisey JG. 2007. The braincase in Paleozoic symmoriiform and cladoselachian sharks. Bulletin of the American Museum of Natural History 307:1-122.

Maisey JG, Miller R, Turner S. 2009. The braincase of the chondrichthyan *Doliodus* from the Lower Devonian Campbellton Formation of New Brunswick, Canada. Acta Zoologica 90:109-122.

Miles RS. 1967. Observations on the ptyctodont fish, *Rhamphodopsis* Watson. Zoological Journal of the Linnean Society 47:99-120.

Miles RS. 1968. Jaw articulation and suspension in *Acanthodes* and their significance. In: Ørvig T, editor. Current Problems of Lower Vertebrate Phylogeny. Nobel Symposium 4. Stockholm: Almqvist & Wiksell. p 109-127.

Miles RS. 1971. The Holonematidae (placoderm fishes), a review based on new specimens of *Holonema* from the Upper Devonian of western Australia. Philosophical Transactions of the Royal Society of London, Series B 263:101-234.

Miles RS. 1973a. Articulated acanthodian fishes from the Old Red Sandstone of England, with a review of the structure and evolution of the acanthodian shoulder-girdle. Bulletin of the British Museum (Natural History), Geology 24:111-213.

Miles RS. 1973b. Relationships of acanthodians. In: Greenwood PH, Miles RS, Patterson C, editors. Interrelationships of Fishes. London: Academic Press. p 63-103.

Miles RS, Westoll TS. 1968. The placoderm fish *Coccosteus cuspidatus* Miller ex Agassiz from the Middle Old Red Sandstone of Scotland. Part I. descriptive morphology. Transactions of the Royal Society of Edinburgh: Earth Sciences 67:373-476.

Miles RS, Young GC. 1977. Placoderm interrelationships reconsidered in the light of new ptyctodontids from Gogo, Western Australia. In: Andrews SM, Miles RS, Walker AD, editors. Problems in Vertebrate Evolution. London: Academic Press. p 123-198.

Miller RF, Cloutier R, Turner S. 2003. The oldest articulated chondrichthyan from the Early Devonian peroid. Nature 425:501-504.

Moy-Thomas JA. 1935. The structure and affinities of *Chondrenchelys problematica* Tr. Proceedings of the Zoological Society of London 105:391-404.

Moy-Thomas JA. 1936. On the structure and affinities of the Carboniferous Cochliodont *Helodus simplex*. Geological Magazine 73:488-503.

Newman MJ, Davidson RG, Den Blaauwen JL, Burrow CJ. 2011. The Early Devonian acanthodian *Euthacanthus gracilis* from the Midland Valley of Scotland. Scottish Journal of Geology 47:101-111.

Ørvig T. 1967a. Phylogeny of tooth tissues: evolution of some calcified tissues in early vertebrates. In: Miles A, editor. Structural and chemical organization of teeth. New York: Academic Press. p 45-110.

Ørvig T. 1967b. Some new acanthodian material from the lower Devonian of Europe. Zoological Journal of the Linnean Society 47:131-153.

Ørvig T. 1975. Description, with special reference to the dermal skeleton, of a new radotinid arthrodire from the Gedinnian of Arctic Canada. In: Lehman JP, editor. Problèmes actuels de Paléontologie-Evolution des Vertébrés. Paris: Colloques Internationaux du Centre National de la Recherche Scientifique. p 41-71.

Otto M. 1991. Zur systematischen Stellung der Lophosteiden (Obersilur, Pisces inc. sedis).

Paläontologische Zeitschrift 65:345-350.
P'an K, Wang S-T. 1978. Devonian Agnatha and Pisces of South China. In: Institute of Geology and Mineral Resources, the Chinese Academy of Geological Sciences, editor. Symposium on the Devonian System of South China. Beijing: Geological Press. p 298-333.
Pan J, Huo F-C, Cao J-X, Gu Q-C, Liu S-Y, Wang J-Q, Gao L-D, Liu C. 1987. Continental Devonian System of Ningxia and its Biotas. Beijing: Geological Publishing House.
Pan J, Wang S-T, Liu S-Y, Gu Q-C, Jia H. 1980. Discovery of Devonian *Bothriolepis* and *Remigolepis* in Ningxia. Acta Geologica Sinica 3:175-185.
Pearson DM. 1982. Primitive bony fishes, with especial reference to *Cheirolepis* and palaeonisciform actinopterygians. Zoological Journal of the Linnean Society 74:35-67.
Pearson DM, Westoll TS. 1979. The Devonian actinopterygian *Cheirolepis* Agassiz. Transactions of the Royal Society of Edinburgh: Earth Sciences 70:337-399.
Pradel A, Maisey JG, Tafforeau P, Janvier P. 2009. An enigmatic gnathostome vertebrate skull from the Middle Devonian of Bolivia. Acta Zoologica 90:123-133.
Qiao T, Zhu M. 2010. Cranial morphology of the Silurian sarcopterygian *Guiyu oneiros* (Gnathostomata: Osteichthyes). Science China Earth Sciences 53:1836-1848.
Qu Q-M, Zhu M, Wang W. 2013. Scales and dermal skeletal histology of an early bony fish *Psarolepis romeri* and their bearing on the evolution of rhombic scales and hard tissues. PloS One 8:e61485.
Qu Q-M, Haitina T, Zhu M, Ahlberg PE. 2015. New genomic and fossil data illuminate the origin of enamel. Nature 526:108-111.
Qu Q-M, Zhu M, Li G. 2010. Synchrotron radiation X-ray microtomography reveals the primitive histological architecture of osteichthyan scales. In: Abstracts of Third International Palaeontological Congress. London: IPC3 Congress Organising Committees.
Rayner DH. 1951. On the cranial structure of an early palaeoniscid, *Kentuckia* gen. nov. Transactions of the Royal Society of Edinburgh: Earth Sciences 62:58-83.
Ritchie A. 1973. *Wuttagoonaspis* gen. nov., an unusual arthrodire from the Devonian of Western New South Wales, Australia. Palaeontographica Abteilung A 143:58-72.
Ritchie A. 1975. *Groenlandaspis* in Antarctica, Australia and Europe. Nature 254:569-573.
Ritchie A. 2005. *Cowralepis*, a new genus of phyllolepid fish (Pisces, Placodermi) from the Late Middle Devonian of New South Wales, Australia. Proceedings of the Linnean Society of New South Wales 126:215-259.
Ritchie A, Wang S, Young GC, Zhang G. 1992. The Sinolepidae, a family of antiarchs (placoderm

fishes) from the Devonian of South China and eastern Australia. Records of the Australian Museum 44:319-370.

Romer AS. 1964. The braincase of the Paleozoic elasmobranch *Tamiobatis*. Bulletin of the Museum of Comparative Zoology 131:87-105.

Schaeffer B. 1981. The xenacanth shark neurocranium, with comments on elasmobranch monophyly. Bulletin of the American Museum of Natural History 169:1-66.

Schultze H-P. 1968. Palaeoniscoidea-schuppen aus dem Unterdevon Australiens und Kansas und aus dem Mitteldevon Spitzbergens. Bulletin of the British Museum (Natural History), Geology 16:343-368.

Schultze H-P. 1973. Crossopterygier mit heterozerker Schwanzflosse aus dem Oberdevon Kanadas, nebst einer Beschreibung von Onychodontida-Resten aus dem Mitteldevon Spaniens und aus dem Karbon der USA. Palaeontographica Abteilung A 143:188-208.

Schultze H-P. 1992. Early Devonian actinoptergians (Osteichthyes, Pisces) from Siberia. In: Mark-Kurik E, editor. Fossil Fishes as Living Animals. Tallinn: Academy of Sciences of Estonia. p 233-242.

Schultze H-P, Cumbaa SL. 2001. *Dialipina* and the characters of basal actinopterygians. In: Ahlberg PE, editor. Major Events in Early Vertebrate Evolution: Palaeontology, Phylogeny, Genetics and Development. London: Taylor & Francis. p 315-332.

Schultze H-P, Märss T. 2004. Revisiting *Lophosteus* Pander 1856, a primitive osteichthyan. Acta Universitatis Latviensis 674:57-78.

Schultze H-P, Zidek J. 1982. Ein primitiver Acanthodier (Pisces) aus dem Unterdevon Lettlands. Paläontologische Zeitschrift 56:95-105.

Soler-Gijón R. 1999. Occipital spine of *Orthacanthus* (Xenacanthidae, Elasmobranchii): structure and growth. Journal of Morphology 242:1-45.

Stensiö E. 1969. Elasmobranchiomorphi Placodermata Arthrodires. In: Piveteau J, editor. Traité de Paléontologie. Paris: Masson. p 71-692.

Stensiö EA. 1925. On the head of the macropetalichthyids with certain remarks on the head of the other arthrodires. Geological Series 4:87-197.

Stensiö EA. 1963. Anatomical studies on the arthrodiran head. Part 1. Preface, geological and geographical distribution, the organization of the head in the Dolichothoraci, Coccosteomorphi and Pachyosteomorphi. Taxonomic appendix. Kungliga Svenska Vetenskapsakademiens Handlingar 9:1-419.

Taverne L. 1997. *Osorioichthys marginis*, "Paléonisciforme" du Famennien de Belgique et la

phylogénie des Actinoptérygiens dévoniens (Pisces). Bulletin de l'Institut royal des Sciences naturelles de Belgique, Sciences de la Terre 67:57-78.

Traquair RH. 1888. Notes of the nomenclature of the fishes of the Old Red Sandstone of Great Britain. Geological Magazine 5:507-517.

Trinajstic K, Boisvert C, Long J, Maksimenko A, Johanson Z. 2015. Pelvic and reproductive structures in placoderms (stem gnathostomes). Biological Reviews 90:467-501.

Turner S, Burrow CJ, Warren A. 2005. *Gyracanthides hawkinsi* sp. Nov. (Acanthodii, Gyracanthidae) from the Lower Carboniferous of Queensland, Australia, with a review of gyracanthid Taxa. Palaeontology 48:963-1006.

Valiukevicius J. 1992. First articulated *Poracanthodes* from the Lower Devonian of Severnaya Zemlya. In: Mark-Kurik E, editor. Fossil Fishes as Living Animals. Tallinn: Academy of Sciences of Estonia. p 193-214.

Warren A, Currie BP, Burrow C, Turner S. 2000. A redescription and reinterpretation of *Gyracanthides murrayi* Woodward 1906 (Acanthodii, Gyracanthidae) from the Lower Carboniferous of the Mansfield Basin, Victoria, Australia. Journal of Vertebrate Paleontology 20:225-242.

Watson DMS. 1937. The acanthodian fishes. Philosophical Transactions of the Royal Society of London, Series B 228:49-146.

Watson DMS. 1938. On *Rhamphodopsis*, ptyctodont from the Middle Devonian Old Red Sandstone of Scotland. Transactions of the Royal Society of Edinburgh: Earth Sciences 59:397-410.

White EI. 1978. The larger arthrodiran fishes from the area of Burrinjuck Dam, N.S.W. Transactions of the Zoological Society of London 34:149-262.

White EI, Toombs HA. 1972. The buchanosteid arthrodires of Australia. Bulletin of the British Museum (Natural History) 22:379-419.

Williams ME. 1998. A new specimen of *Tamiobatis vetustus* (Chondrichthyes, Ctenacanthoidea) from the Late Devonian Cleveland Shale of Ohio. Journal of Vertebrate Paleontology 18:251-260.

Woodward AS. 1924. On a hybodont shark (*Tristychius*) from the Calciferous Sandstone Series of Eskdale (Dumfriesshire). Quaternary Journal of Geological Society 80:338-342.

Woodward AS, White EI. 1938. The dermal tubercles of the Upper Devonian shark, Cladoselache. Annals and Magazine of Natural History 2:367-368.

Young GC. 1978. A new Early Devonian petalichthyid fish from the Taemas/Wee Jasper region of New South Wales. Alcheringa 2:103-116.

Young GC. 1979. New information on the structure and relationships of *Buchanosteus* (Placodermi: Euarthrodira) from the Early Devonian of New South Wales. Zoological Journal of the Linnean Society 66:309-352.

Young GC. 1980. A new Early Devonian placoderm from New South Wales, Australia, with a discussion of placoderm phylogeny. Palaeontographica Abteilung A 167:10-76.

Young GC. 1986a. Relationships between northern and southern vertebrate faunas during the Middle Palaeozoic. In: McKenzie KG, editor. Shallow Tethys 2. Wagga Wagga: A. A. Balkema. p 79-85.

Young GC. 1986b. The relationships of placoderm fishes. Zoological Journal of the Linnean Society 88:1-57.

Young GC. 1989. New occurrences of culmacanthid acanthodians (Pisces, Devonian) from Antarctica and southeastern Australia. Proceedings of the Linnean Society of New South Wales 111:12-25.

Young GC, Goujet D. 2003. Devonian fish remains from the Dulcie Sandstone and Cravens Peak Beds, Georgina Basin, central Australia. Records of the Western Australian Museum Supplement 65:1-85.

Yu X-B. 1998. A new porolepiform-like fish, *Psarolepis romeri*, gen. et sp. nov. (Sarcopterygii, Osteichthyes) from the Lower Devonian of Yunnan, China. Journal of Vertebrate Paleontology 18:261-274.

Zangerl R, Case GR. 1976. *Cobelodus aculeatus* (Cope), an snacanthous shark from Pennsylvanian black shales of North America. Palaeontographica Abteilung A 154:107-157.

Zhang G-R, Wang J-Q, Wang N-Z. 2001. The structure of pectoral fin and tail of Yunnanolepidoidei, with a discussion of the pectoral fin of chuchinolepids. Vertebrata PalAsiatica 39:9-19.

Zhang M-M. 1980. Preliminary note on a Lower Devonian antiarch from Yunnan, China. Vertebrata PalAsiatica 18:179-190.

Zhu M. 1991. New information on *Diandongpetalichthys* (Placodermi: Petalichthyida). In: Chang M-M, Liu Y-H, Zhang G-R, editors. Early Vertebrates and Related Problems of Evolutionary Biology. p 179-194.

Zhu M. 1996. The phylogeny of the Antiarcha (Placodermi, Pisces), with the description of Early Devonian antiarchs from Qujing, Yunnan, China. Bulletin du Muséum national d'Histoire naturelle 18:233-347.

Zhu M, Ahlberg PE. 2004. The origin of the internal nostril of tetrapods. Nature 432:94-97.

Zhu M, Schultze H-P. 1997. The oldest sarcopterygian fish. Lethaia 30:293-304.

Zhu M, Schultze H-P. 2001. Interrelationships of basal osteichthyans. In: Ahlberg P, editor. Major Events in Early Vertebrate Evolution: Palaeontology, Phylogeny, Genetics and Development. London: Taylor & Francis. p 289-314.

Zhu M, Yu X-B. 2002. A primitive fish close to the common ancestor of tetrapods and lungfish. Nature 418:767-770.

Zhu M, Yu X-B. 2004. Lower jaw character transitions among major sarcopterygian groups - a survey based on new materials from Yunnan, China. In: Arratia G, Wilson MVH, Cloutier R, editors. Recent Advances in the Origin and Early Radiation of Vertebrates. München: Verlag Dr. Friedrich Pfeil. p 271-286.

Zhu M, Yu X-B. 2009. Stem sarcopterygians have primitive polybasal fin articulation. Biology Letters 5:372-375.

Zhu M, Yu X-B, Choo B, Qu Q-M, Jia L-T, Zhao W-J, Qiao T, Lu J. 2012a. Fossil fishes from China provide first evidence of dermal pelvic girdles in osteichthyans. PloS One 7:e35103.

Zhu M, Yu X-B, Janvier P. 1999. A primitive fossil fish sheds light on the origin of bony fishes. Nature 397:607-610.

Zhu M, Yu X-B, Wang W, Zhao W-J, Jia L-T. 2006. A primitive fish provides key characters bearing on deep osteichthyan phylogeny. Nature 441:77-80.

Zhu M, Yu X-B, Ahlberg PE, Choo B, Lu J, Qiao T, Qu Q-M, Zhao W-J, Jia L-T, Blom H, Zhu Y-A. 2013. A Silurian placoderm with osteichthyan-like marginal jaw bones. Nature 502:188-193.

Zhu M, Yu X-B, Choo B, Wang J, Jia L-T. 2012b. An antiarch placoderm shows that pelvic girdles arose at the root of jawed vertebrates. Biology Letters 8:453-456.

Zhu M, Zhao W-J, Jia L-T, Lu J, Qiao T, Qu Q-M. 2009. The oldest articulated osteichthyan reveals mosaic gnathostome characters. Nature 458:469-474.
